# Membrane-Sensitive Conformational States of Helix 8 in the Metabotropic Glu2 Receptor, a Class C GPCR

**DOI:** 10.1371/journal.pone.0042023

**Published:** 2012-08-01

**Authors:** Agostino Bruno, Gabriele Costantino, Gianni de Fabritiis, Manuel Pastor, Jana Selent

**Affiliations:** 1 Computer-Assisted Drug Design Lab, Research Programme on Biomedical Informatics (GRIB), Universitat Pompeu Fabra, Barcelona, Spain; 2 Pharmaceutical Department, University of Parma, Parma, Italy; 3 Computational Biochemistry and Biophysics Laboratory, Research Programme on Biomedical Informatics (GRIB), Universitat Pompeu Fabra, Barcelona, Spain; University of Oldenburg, Germany

## Abstract

The recent elucidation of the X-ray structure of several class A GPCRs clearly indicates that the amphipathic helix 8 (H8) is a conserved structural domain in most crystallized GPCRs. Very little is known about the presence and the possible role of an analogous H8 domain in the distantly related class C GPCRs. In this study, we investigated the structural properties for the H8 domain of the mGluR2 receptor, a class C GPCR, by applying extended molecular dynamics simulations. Our study indicates that the amphipathic H8 adopts membrane-sensitive conformational states, which depend on the membrane composition. Cholesterol-rich membranes stabilize the helical structure of H8 whereas cholesterol-depleted membranes induce a disruption of H8. The observed link between membrane cholesterol levels and H8 conformational states suggests that H8 behaves as a sensor of cholesterol concentration.

## Introduction

Recent years have seen an increasing availability of experimentally determined 3D structures of class A, rhodopsin-like, G Protein Coupled Receptors (GPCRs), allowing a better understanding of their mechanism of activation at a molecular level [1], [2]. Much less is known about the distantly related class C GPCRs, for which no experimentally determined structure of the transmembrane domain is available yet. Class C include metabotropic glutamate receptors, GABA_B_ receptors, sweet and umami taste receptors and the calcium-sensing receptor. Class C GPCRs are characterized by an unusually extended N-terminal domain, which is structurally ordered and contains the binding site for orthosteric ligands. Another characteristic feature of class C GPCRs is their constitutive dimerization, both as homo- and heteromers [3]–[5]. In common with class A receptors, class C GPCRs have a transmembrane domain composed of seven hydrophobic stretches, transducing signals from the extracellular to the intracellular space [4], [6]. Despite the low sequence similarity, several lines of evidence support the hypothesis that class C GPCRs have a heptahelical transmembrane domain (7TM) similar to that of class A, and also a similar mechanism of activation and coupling to G proteins [4], [6], [7]. In particular, signatures for 7TM activation such as an ionic lock between TM3 and TM6 and the W^6.50^ and the Y^7.53^ are also present in class C GPCRs. Furthermore, it is particularly relevant that truncated class C GPCRs, lacking both the N-terminal domain and the intracellular C-tail can be activated by allosteric modulators acting at a binding site localized in the transmembrane domain and conserved with the binding pocket of class A GPCRs [6]–[8]. Finally, several site-directed mutagenesis experiments designed on the basis of 3D models of class C GPRCs were found to be predictive [9], [10]. Taken together, these observations suggest that 3D models of the heptahelical transmembrane domain of class C GPCRs based on class A receptors can have heuristic value and be of use for deciphering aspects related to their mechanism of activation.

The class C Metabotropic glutamate receptor type 2 (mGluR2) has received attention as a potential drug target [11]–[15] and has been recently described to be involved in the heteromerization with the class A 5-HT_2A_ serotonin receptors [11], [14], [15]. In the framework of a project aimed at understanding the structural basis and the pharmacological relevance of such heteromerization [16], [17], we became interested in studying how reliable is the 3D structure corresponding to the transmembrane portion of this receptor. In class A GPCRs, an increasingly recognized feature is the presence of a relatively short amphipathic domain, termed helix 8 (H8) located immediately after the end of the seventh transmembrane domain (TM) towards the cytoplasmatic tail [18]. The recent elucidation of the x-ray structure of several class A GPCRs clearly identifies H8 as a conserved structural domain which folds as an α-helix in most GPCRs so far crystallized [19]–[27]. Due to its amphipathic character, H8 preferably adopts a location parallel to the membrane plane, in a polar/hydrophobic interface between membrane and the cytoplasmic side of the cell. The apparent conservation of H8 among class A GPCRs and its crucial position towards the intracellular side where G-proteins coupling occurs, suggest that this domain can play an important functional role [18]. Thus, H8 has been proposed to be involved in G-protein coupling and activation [18], [28], in GPCR expression and trafficking [29], [30], in GPCR internalization [30], and dimerization [31]–[33]. In this respect, the conformational properties of the H8 domain in different GPCR families have attracted, recently, considerable attention [29], [34]–[38].

In contrast, very little is known about the presence and the possible role of an analogous H8 domain in the distantly related class C GPCRs, although several lines of evidence support its existence [5], [39]–[41]. Early observations indicate that the carboxy terminus of mGluRs is involved in direct coupling with G-proteins [5], [6], and that H8 (full length of the putative H8 817 ILFQPQKNVVSHRAPTS 834), the so-called fourth intracellular loop, may have some helicity. There are also reasons for questioning the presence of a stable H8: (i) the degree of similarity between mGluRs and class A GPCR is very low in the predicted region of H8 [5], [42]; (ii) mGluRs, and mGluR2 in particular, lack conserved motifs so far assigned to the H8 [42]; (iii) the predicted amphipathicity and helicity of the carboxy tail of the seventh transmembrane domain of mGluR2 is in the twilight zone of statistical significance [39].

In this scenario, elucidating the, so far elusive, structural features of H8 in class C GPCRs, and mGluR2 in particular, can advance our knowledge about class C GPCR functionality and provide new insights in the structure of this important class of drug targets. In cases like this, computational techniques have proven to produce useful results [43]–[48]. In the present work, we take advantage of modern computational approaches to simulate structural features of H8 in mGluR2. Thus, we set up a computational study of the transmembrane domain of mGluR2 involving extended molecular dynamics (MD) simulations, for a total simulation time >3.5 µs, in an explicit membrane environment of 1-sterayol-2-docosahexaenoyl-phosphatidylcholine (SDPC) at different cholesterol concentrations: 0% (system 1) and 25% (system 2). Simulations for both systems were performed using the ACEMD software [49], and CHARMM force field implemented with parameters (http://mackerell.umaryland.edu/CHARMM_ff_params.html) for cholesterol and SDPC molecules. Our obtained results strongly support the existence of an α-helical structured H8 in mGluR2 but only under certain conditions, tightly linked to the membrane composition. This study reveals that cholesterol, an important component in cell membranes, drives H8 stabilization by both direct and indirect effects. In the absence of membrane cholesterol, H8 loses its defined α-helical structure adopting an ensemble of different destabilized conformational states. All in all, these results suggest that mGluR2-H8 adopts membrane-sensitive conformational states, thus behaving as a “sensor of cholesterol concentration”.

## Results

### Conformational Analysis of the mGluR2-Helix 8 in Different Membrane Environments

A general assessment of the dynamic behavior of the mGluR2 in the presence (system 1) and in the absence of cholesterol (system 2) shows stable transmembrane regions and, as expected, more flexible intra- and extracellular loop regions (IL1-3 and EL1-3) for both systems (Figure S1A). However, it is intriguing that H8 becomes unstable and displays increased flexible features when evolving in a cholesterol-depleted membrane environment (Movie S1). In order to rule out that the effect of the membrane composition on H8 stability is due to a random effect in our simulations, we performed a statistical analysis based on 10 MD runs for both systems (total simulation time 3.2 µs). A visual inspection of the 10 MD runs of the mGluR2 receptor in cholesterol-rich membranes reveals in all cases an integer α-helical H8 structure (100% folded) (Table S1). Importantly, the 10 MD runs in the cholesterol-depleted membrane show a destabilization of H8 in 60% of the runs, whereas H8 maintains folded in 40% of the runs (Table S1).

**Figure 1:**
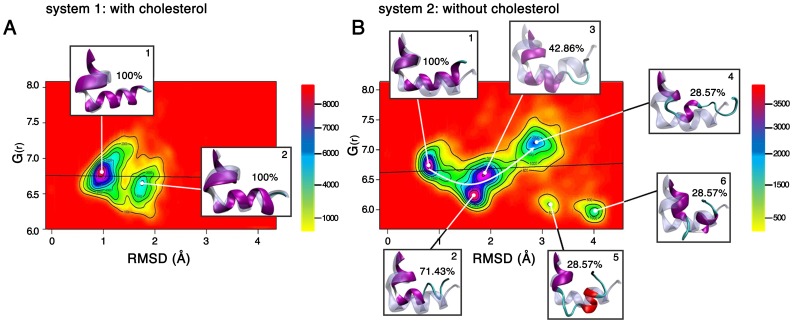
Probability Density Functions (PDF) of the collected simulations with and without cholesterol. PDF plot reflecting the conformational space of H8 in a cholesterol-rich (A) and in a cholesterol-depleted (B) membrane system computed over 10 MD-runs each. The colored bar beside the PDF plot describes the density estimation. The average structure of each cluster (cartoon and purple) is depicted in the insets and superimposed to the initial state of H8 (cartoon and transparent light blue). The amount of integer α-helical structure is expressed as percentage of hydrogen bonds formed by backbone atoms which stabilize the α-helical structure.

With the aim to shed light on the structural bases of the different conformational H8 behavior in the presence (system 1) and absence (system 2) of cholesterol, we concatenated the 10 MD runs for each system and estimated a probability density function (PDF) using a two dimensional space defined by the values of the radius of gyration (G_(r)_) and the root-mean square deviations (RMSD) of the H8 from the starting structure. Such PDF plots allow distinguishing between different probable H8 states, which occur in cholesterol-rich (Figure 1A) and cholesterol-depleted (Figure 1B) systems during the accumulated simulation time of 1.6 µs each. The PDF plots highlight significant differences in conformational states of mGluR2-H8 and their probabilities between the cholesterol-rich (Figure 1A) and cholesterol-depleted (Figure 1B) receptor-membrane systems. In the cholesterol-rich system, the mGluR2-H8 is characterized by two states (Figure 1A). State 1 has a higher probability than state 2; both contain an intact 100% folded α-helical structure as indicated by the average structure of each cluster (Figure 1A, insets). In contrast, in the cholesterol-depleted system (Figure 1B), the mGluR2-H8 adopts multiple conformational states with the following probability: states 1–3 (high), state 4 (medium), state 5 and 6 (low). Among them, state 1 is 100% folded as reflected by the average structure (Figure 1B, inset) and similar to the one found in state 1 of the cholesterol-rich simulation (Figure 1A, inset). A partial destabilization of the α-helical H8 structure is seen for state 2 and 3 of the cholesterol-depleted systems (Figure 1B, inset, state 2: 71.43% folded and state 3: 42.86% folded). Finally, a strongly disturbed H8 containing only 28.57% of the original α-helical starting structure is found in state 4 (Figure 1B, inset). According to the PDF plot of the cholesterol-depleted system (Figure 1B), the highly probable states 2 and 3 could represent transition states between the completely folded state 1 and the strongly disturbed H8 in state 4. The slightly separated and less probable states 5 and 6 show also a strongly destabilized H8 (28.57% folded) and may originate from state 4 (Figure 1B and insets). The results of the conformational analysis of mGluR2-H8 using PDF plots for 3.2 µs accumulated simulation time, strongly suggests that the presence/absence of cholesterol drives the conformational state of H8: thereby, cholesterol presence stabilizes the canonical amphipathic mGluR2-H8 whereas its absence has a destabilizing effect.

### A Cholesterol-Dependent Mechanism Drives the H8 Conformational Flexibility

The observed cholesterol-dependent stabilization/destabilization of the α-helical H8 structure raises the question about what is the underlying molecular mechanism. The analysis of the individual 160 ns production runs of the mGluR2 embedded in a cholesterol-rich membrane reveals direct and indirect cholesterol effects as crucial determinants for H8 stabilization. Direct cholesterol contacts are mediated by at least two cholesterol molecules which occupy a pocket formed by TM1, TM7 and H8 (Figure S2 and S3) interacting steadily with hydrophobic and polar residues of the α-helical structure of H8 during the 160 ns (Figure S2 and S3). These firm cholesterol contacts most likely promote a proper H8 location with respect to the polar head groups of the SDPC molecules, and a stable interaction between the H8 and the membrane layer (Figure S2 and S3), thus contributing to a stable H8 domain. This finding is consistent with the fact that direct cholesterol-GPCR contacts have been reported to play crucial role in the stabilization of the secondary structure of different GPCRs [50], [51].

**Figure 2:**
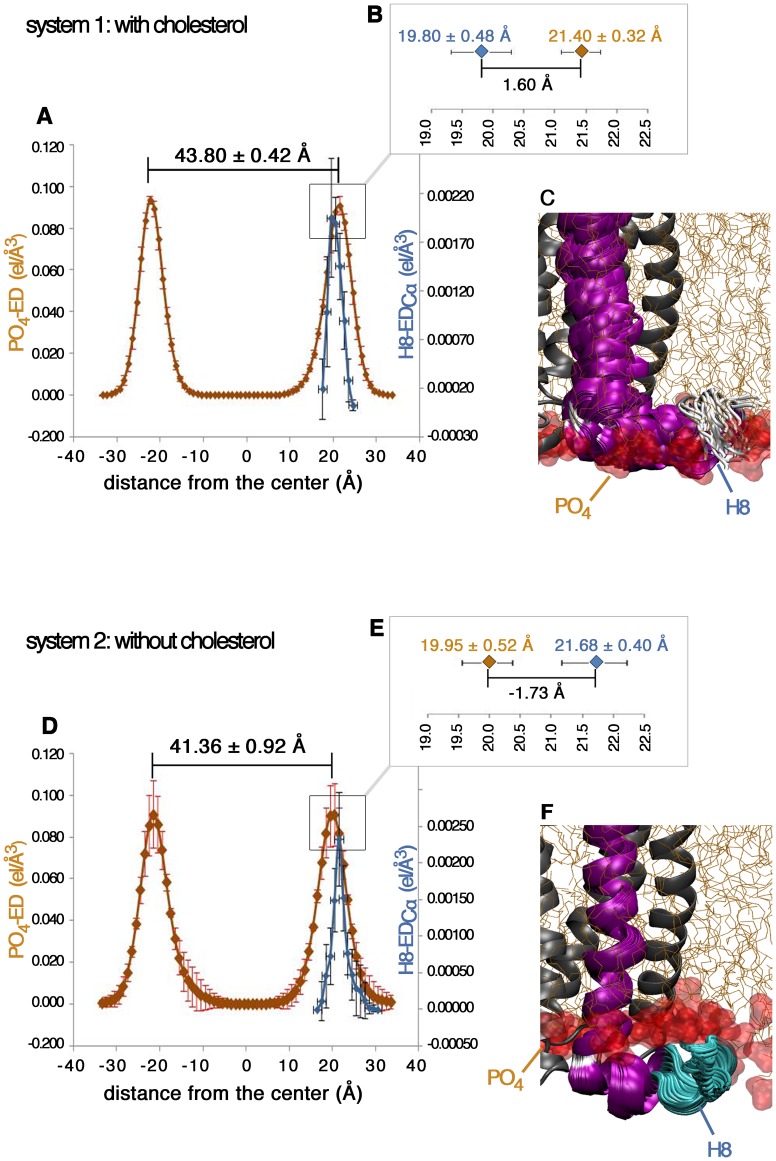
Effect of cholesterol-driven membrane thickness on H8 location. Electron density (ED) of the PO_4_ groups (brown line) and H8 (blue line) for the simulation with (A, B) and without (D, E) cholesterol, the red and black bars refer to the standard deviation for each slab of the PO_4_- ED and H8-ED_Cα_ values. (C) Location of H8 in a cholesterol-rich system relative to the PO_4_ groups corresponding to state 1 (see Figure 1A) showing each 10th frame of a total 981 frames. (F) Location of H8 in a cholesterol-depleted system relative to the PO_4_ groups corresponding to state 4 (see Figure 1B) showing each frame of a total 51 frames.

However, the most interesting finding is the indirect cholesterol effect on the mGluR2-H8 stability mediated by the thickness of the membrane bilayer. Plotting the average electron density (ED) profile of the membrane bilayer for all runs with and without cholesterol shows a consistent cholesterol-depended increase in membrane thickness (Figure 2A and D). The systems with cholesterol adopt an average peak-to-peak distance (equal to bilayer thickness) of 43.8±0.42 Å (brown line, Figure 2A) in which each ED peak refers to the PO_4_ group of the SDPC membrane (D_PO4–PO4_). This is in good agreement with experimental and computational values available in literature [52]–[55]. The system without cholesterol (brown line, Figure 2D) shows an approx. 2 Å smaller D_PO4–PO4_ distance (41.36±0.92) than the simulation with cholesterol (Figure 2A). The physical correctness of the observed cholesterol-mediated increase in membrane thickness is also supported by measuring the peak-to-peak distance for carbonyl groups of the lipid tails: with cholesterol: 35.00±0.63 Å, without cholesterol: 30.00±1.15 Å (Figure S6) which are once more in good agreement with experimental data [52]–[55].

The role of the bilayer thickness in the definition of the H8 conformational state can be further evinced by plotting the average location of the hydrophobic face of the amphipathic H8 (residues Q821, K822, V824, V825, S826, R828, and A829, Figure S2B) as ED of the Cα atoms (H8-ED_Cα_, blue line, Figure 2A,D) over the average membrane thickness as ED of the PO_4_ group of the SDPC membrane (PO_4_-ED, brown line, Figure 2A,D). Thereby, the H8-ED_Cα_ and the PO_4_-ED are averaged over 1.6 µs for each system. In the simulation with cholesterol, the peak of the hydrophobic H8 face is located 1.60 Å left from the peak of the PO_4_ groups (blue line, Figure 2A) whereas in the simulation without cholesterol the hydrophobic face of H8 (blue line, Figure 2D) is shifted to 1.73 Å right from the PO_4_ groups (blue line, Figure 2D). The visual inspection (Figure 2C and F) of the observed cholesterol-dependent H8 shift (total difference of 3.33 Å) suggests a possible mechanism of how cholesterol indirectly drives H8 stabilization via membrane thickness. In the cholesterol rich system, the H8 is nicely embedded in the membrane, exposing the hydrophobic H8 face to the hydrophobic aliphatic chains and the cholesterol molecules. The polar H8 face contacts the polar PO_4_ groups (Figure 2C) and is partially solvent-exposed. In contrast, in the cholesterol-depleted system, H8 drops out of the membrane bilayer into a completely polar environment due to the smaller membrane thickness (Figure 2F). As a result, H8 looses important hydrophobic contacts of its hydrophobic face with membrane aliphatic chains. This leads to a destabilized and more flexible H8 with various conformational states as found in cluster 2, 3, 4 and 5 (Figure 1B) for the cholesterol-depleted system, supporting the hypothesis that a correct orientation of the mGluR2-H8 hydrophobic face in the membrane environment is crucial for the definition of the H8 conformational state. These findings point out, for the first time the existence of different membrane-sensitive conformational states for the mGluR2-H8 which could behave as a sensor of cholesterol concentration.

### Helix 8 – A Real Structural Element in the mGluR2

In order to further assess the existence of an α-helical H8 structure in the mGluR2, we carried out a structural prediction analysis. As a first approach, the amphipathic character of the putative mGluR2 H8 was assessed by AmphipaSeek (http://npsa-pbil.ibcp.fr/cgi-bin/npsa_automat.pl?page=/NPSA/npsa_amphipa-seek.html), along with an evaluation of the potential presence of IPM anchor points, and the hydrophobic moment, but this method failed to predict successfully some validation structures, including bovine rhodopsin and the chemokine receptor (Experimental Procedures S1).

Using an alternative approach, we screened the Protein Data Bank using BLAST (http://blast.ncbi.nlm.nih.gov/Blast.cgi) for sequence homologues to the mGluR2-H8 sequence (ILFQPQKNV) (Table 1). In a first step, we validated the approach using H8 sequences of the bovine rhodopsin (complete α-helical H8) and the CXCR4 receptors (coil structure with a small α-helical portion, 3ODU) with known three-dimensional structure. For the CXCR4, receptor we used the H8 sequence reported in the x-ray crystal structure, where Phe and Leu residues were mutated to two Ala residues [23]. Only the three first hits that were not X-ray crystal structures of the same receptor (Table 1) were taking into account for this validation step. Applying this protocol, we retrieved 3 protein structures (PDB IDs 3QGB, 3K5Q, 1LKX) for the bovine rhodopsin (Table 1). All three protein structures reveal an α-helical structure, thus successfully predicting an α-helical structure for the H8 which is in accordance with the X-ray structure of bovine rhodopsin receptor. More intriguing, for the CXCR4 receptor, we retrieved 3 protein structures (PDB IDs 1SJ8, 2JRC, 2BEK) which reflect well the short region with an α-helical structure AQHAL (Table 1, PDB IDs 1SJ8 and 2BEK) but also the partially misfolded region of H8 (Table 1, PDB ID 2JRC). After the successful validation step, the same protocol was applied to the corresponding H8 sequence for the mGluR2 receptor (Table 1). The analysis revealed three protein structures (PDB IDs 3EFO, 1IC8, 2I5D, Table 1) with a homologue sequence fragment exhibiting α-helical features point to a real α-helical mGluR H8 domain.

**Table 1:** Prediction of the secondary structure of the H8 domain for bovine rhodopsin, the chemokine CXCR4 and the mGluR2 receptor.

	Related Protein	Related Sequences	Secondary Structure	Score (bits)
**Bovine rhodopsin**307 **IMMNKQFRNCMVTTLCCGK** 325	**3QGB.pdb**	**Rhod. Bov.** 307 **IMMNKQFRN**––**CMVTTLCC** 323**3QGB** 330 **IMM**FH**QF**G**N**YVVQ**CM**L-**T**I**CC** 349	α-helix	25.7
	**3K5Q.pdb**	**Rhod. Bov.** 307 **IMMNKQFRN**––**CMVTTLCC** 323**3K5Q** 329 **IMM**FH**QF**G**N**YVVQ**CM**L-**T**I**CC** 348	α-helix	25.7
	**1LKX.pdb**	**Rhod. Bov.** 312 **QFRNCM**–-**VTTL** 321 **1LKX** 565 **QFRN**A**M**NALI**TTL** 577	α-helix	24.0
	**Related Protein**	**Related Sequences**	**Secondary Structure**	**Score (bits)**
**CXCR4 receptor**303 AFLGAKFKTS**AQHAL**TSV 320	**1SJ8.pdb**	**CXCR4** 311 TS**AQ** **HAL** T 318 **1SJ8** 8 TS**AQ**Q**AL** T 15	α-helix	23.5
	**2JRC.pdb**	**CXCR4** 305 LGAKFK 310 **2JRC** 36 LGAKFK 41	random coil	21
	**2BEK.pdb**	**CXCR4** 310 KTS**AQHAL** TS 319 **2BEK** 220 KTI**AQHA**PTS 229	α-helix	21
	**Related Protein**	**Related Sequences**	**Secondary Structure**	**Score**
**mGluR2 receptor**817 **ILFQPQKNVVSHR** 829	**3EFO.pdb**	**mGluR2** 817 **ILFQPQKNV** 825 **3EFO** 347 **ILFQ** PQT**NV** 355	Partial α-helix	28.6
	**2I5D.pdb**	**mGluR2** 819 **FQP**––––––-**QKNVVSHR** 829 **2I5D** 158 **FQP**DGYEQTYAEMPKAE**KN**A**VSHR** 181	α-helix	23.1
	**1IC8.pdb**	**mGluR2** 817 **ILFQP**–-**QKN** 824 **1IC8** 129 **ILFQ**AYER**QKN** 139	α-helix	20.6

Numbering from the full length sequences of each protein. Underlined residues are conserved among two sequences. Residues highlighted in bold correspond to an α-helical structure.

## Discussion

In this work, we propose the existence of an amphipathic H8 with an α-helical conformation in class C mGluR2. Extended MD simulations reveal that the dynamic properties of the α-helical H8 are affected by the environment which is in agreement with recently reported data for other GPCRs [29], [34]–[38]. As a matter of fact, an environment-dependent H8 conformation has been also suggested for the CXCR4 receptor by [23]. The crystal structure (PDB 3ODU) exhibits only a small α-helical portion in the H8 domain instead of an earlier assumed canonical α-helical H8 structure. The authors ascribe this finding to two mutations in H8 as well as to crystallization conditions [23].

The results of our study indicate that cholesterol influences the H8 conformational state through direct and indirect effects. Direct cholesterol effects are mediated by an unprecedented cholesterol binding cleft for the mGluR2 receptor (Figure S2 and S3). Similar direct cholesterol contacts have been reported experimentally for other GPCRs [56]. Nevertheless, the most important finding is an indirect effect on the H8 conformational state which takes place by a cholesterol-dependent alteration of the membrane thickness (Figure 2). Noteworthy, the simulated cholesterol effect on membrane thickness is a realistic biophysical effect consistent with experimental data [57]–[60]. Our study predicts that in cholesterol-depleted membranes, the membrane environment is altered and the amphipathic helix 8 drops out of its stabilizing amphipathic environment, thus causing an increase in flexibility and conformational states. Based on the observed link between membrane cholesterol and H8 conformation, we postulate that H8 behaves in mGluR2 as a sensor of cholesterol concentration by adopting different membrane-sensitive conformational states at the C-terminus of the receptor (Figure 1–2). Noteworthy, the C-terminus of mGluRs is most likely involved in direct G-protein coupling [5], [6], [39] and the membrane-sensitive conformational H8 states could be part of a dynamic mechanism to regulate mGluR2 signaling transduction. A similar mechanism has been reported for other GPCRs such as the PAR1 [35], and the rhodopsin receptors [36].

On a molecular level, we suggest for the mGluR2 that H8 exposes different amino acids residues to the intracellular side of the cell in dependence on the membrane-sensitive conformational states. Such transformed intracellular epitope can drive the binding interaction to different intracellular proteins such as G-protein or beta-arrestin, thus dramatically impacting receptor signalling. This exciting possibility is compatible with the GPCR ensemble theory [61], postulating that membrane-bound receptors adopt different micro-conformational states at the cytoplasmic side that regulate different downstream signaling pathways, such as G protein-dependent [35] and -independent pathways [36].

The presence of helix 8 as a real structural element in the mGluR2 is further supported by screening the Protein Data Bank for sequence homologues using BLAST (Table 1). This approach was first successfully validated by predicting the canonical α-helical structure of the rhodopsin H8. Additionally, we were able to exactly predict both the coil and α-helical regions of the mutated CXCR4 H8 sequence (3ODU), stressing the reliability of our protocol. In a second step, the same protocol was applied to the mGluR2 H8 domain predicting an α-helical feature for the H8 domain. This finding corroborates once more that H8 of the mGluR2 is able to adopt an α-helical conformation. However, as indicated by our extended MD simulation, the stable α-helical H8 occurrence is tightly linked to the membrane environment.

All in all, our data allow us to postulate a mechanistic link between cholesterol concentration, membrane properties, H8 stability, and receptor functioning. This postulation presents also a possible connection between evidences that cholesterol is a factor released by glia to modulate membrane properties [57]–[60], and that mGluR2 functioning, and trafficking are tightly linked to the membrane composition [62]–[64]. The elucidation of the precise physiological significance of this potential link cannot be fully anticipated from our results, since the lack of the extended extracellular ligand binding domain in our model prevents a thorough analysis of the coupling between the ligand binding domain and the intracellular portion. Nonetheless, the clear effect of the cholesterol concentration on the structure of H8 adds an important piece of information which can be extremely useful in deciphering the complex mechanisms of receptor activation and signalling in class C GPCRs.

Finally, it is worth to mention that most of the so far reported MD simulations of GPCRs in an explicit membrane environment did not take cholesterol as membrane component into account. In this study, we show that cholesterol plays a crucial role in GPCR’s flexibility and conformational behaviour. Moreover, our results could have an impact not only in the GPCRs field, but also in the study of other membrane-bound proteins, for which similar effects were reported [65]. Therefore, the methodology under investigation may be applicable also to other GPCR families or membrane–bound proteins for which cholesterol effects are reported, constituting a tool of general interest in biomedical and pharmaceutical research.

## Methods

### Homology Modelling of the mGluR2 Receptor

The 3D structure of the mGluR2 receptor was built using the structure of the bovine rhodopsin (1GZM) as template and the sequence of the human mGluR2 receptor (Q14416). The 3D structure of the mGluR2 was generated using the MODELLER software (salilab.org/modeller), and the quality of the model was assessed on the basis of structural properties (Figure S9, S10 and S11). Moreover, to evaluate the structural architecture of the obtained mGluR2 model in more detail, two recently reported mGluR2 negative allosteric modulators, RO4988546 and RO5488608 were docked into the model of TM-mGluR2 (Chart S1 and Figure S12 and Experimental Procedures S1). Importantly, the ligand-receptor interactions are in good agreement with mutagenesis data reported by [10], stressing the biological relevance of mGluR2 model. A complete description about the procedure employed for the generation of the model and validation protocol are provided in the Experimental Procedures S1.

### Generation of the mGluR2-membrane System

Two different pre-equilibrated SDPC phospholipids bilayers were generated using the membrane-builder tool of charm-gui.org (http://www.charmm-gui.org): (*i)* 94×94 Å (xy) 0% of cholesterol; (*ii*) 94×94 Å (xy) with a ratio SDPC:cholesterol equal to 1:3 (Table S2 and Experimental Procedures S1). In order to place the receptor into the bilayer a hole was generated, and lipids in close contact (<1 Å distance from any protein atoms) were deleted. For the membrane with cholesterol some SDPC or cholesterols molecule were manually deleted in each layer in order to retain the same cholesterol concentration (25%). The membrane-receptor complexes thus obtained were solvated and neutralized using the solvation and autoionize modules of VMD1.8.7 [66]. The ionic strength was kept at 0.15 M by NaCl and we used TIP3 water model. The all-atom models of each system were generated by using the CHARMM force-field parameters (http://mackerell.umaryland.edu/CHARMM_ff_params.html). Before the relaxation step each system was submitted to a minimization procedure for 1000 steps.

### Molecular Dynamics Simulations Protocol

During the relaxation phase the system were equilibrated using the NPT ensemble with a target pressure equal to 1.01325 bar, a time-step of 2 fs and using the RATTLE algorithm for the hydrogen atoms. In this stage, the harmonic constraints were progressively reduced until an elastic constant force equal to 0 kcal/mol, and the temperature was increased to 300K (Table S3 and Experimental Procedures S1). All the simulations were conducted using the same non-bonded interaction parameters, with a cutoff of 9 Å, a smooth switching function of 7.5 Å and the non-bonded pair list set to 9.5 Å. The periodic boundary conditions were set using the system size shown in Experimental Procedures S1, and for the long range electrostatics we used the PME methodology with a grid spacing of 1 Å [67]. Each production phase was performed using the same parameters, with a time-step of 4 fs, and a hydrogen scaling factor of 4 (detailed description about the construction of the membrane-receptor complexes, and the MD simulations protocol are provided in the Experimental Procedures S1).

### Probability Density Function and Electron Density Profile

A probability density function (PDF) was estimated using a two dimensional space defined by the values of the radius of gyration (G_(r)_) and the RMSD of the H8 from the starting structure (Figure S4, S5 and Table S4). Kernel Density Estimation (KDE) is a non-parametric way of estimating the PDF of random variable. To compute the PDF we used the Free Statics and Forecasting Software Server, based on R language (http://www.wessa.net/rwasp_bidensity.wasp and Experimental Procedures S1). To quantify the membrane thickness, we computed the local 1-D electron density profile of different species and functional groups (such as the entire SDPC and cholesterol molecules or the PO_4_ groups of the SDPC molecules) projected along the bilayer normal (Figure S6, S13 and Experimental Procedures S1). The density profile computations were performed with the VMD plugin *Density Profile Tool* (http://multiscalelab.org/utilities/DensityProfileTool). Detailed description about Probability Density Function and Electron Density Profile calculations are provided in the Experimental Procedures S1.

### Structural Prediction Analysis of the H8 Domain

The amphipathic character of the putative mGluR2 H8 was assessed by AmphipaSeek (Experimental Procedures S1), along with an evaluation of the potential presence of in-plane membrane (IPM) anchor points, an structural motif of amphipathic helices (Figure S7 and S8). However, AmphipaSeek did not identify any IPMs in mGluR2, human CXCR4, as well as bovine rhodopsin although it is known that bovine rhodopsin has an amphipathic in-plane α-helix, with two palmitoylation sites [68], [69]. Moreover, applying AmphipaSeek for computing the amphipathicity and the hydrophobic moment yielded only inconsistent patterns when comparing GPCRs with/without conserved H8 (Figure S8). Hence, AmphipaSeek is not a suitable tool for detecting amphipathic H8 domains in GPCRs.

To support the genuine occurrence of an α-helical H8, we screened the Protein Data Bank using BLAST (http://blast.ncbi.nlm.nih.gov/Blast.cgi) for sequences homolog to the mGluR2-H8 sequence (ILFQPQKNV) (Table 1). In a first step, we validated our search protocol by using the corresponding H8 sequences of the bovine rhodopsin and CXCR4 receptors (Table 1) taking into account only the first three proteins that were retrieved by our search protocol and that were not X-ray crystal structures of the same receptor (Table 1). In a second step, the same search protocol was applied to the corresponding H8 sequence for the mGluR2 receptor (Table 1).

## Supporting Information

Figure S1 **Typical average RMSD of the mGluR2. (A)** Average RMSD per residue of the Cα atoms of the mGluR2 receptor. The blue line refers to the simulation with cholesterol (25%), the red one refers to the simulation without cholesterol (0%). The regions highlighted in grey represent the transmembrane regions (TM1–7) and the Helix 8 (H8). (**B**) RMSD of Cα atoms of mGluR2 with (blue) and without (brown) cholesterol for a single MD run.
(DOCX)

Figure S2 **Representation of the putative cholesterol pocket.** Representation of direct cholesterol contacts with the mGluR2 receptor. (**A–B**) Different views of the cholesterol cleft described by TM1-TM7-H8 (new cartoon and purple, and transparent and white) the whole receptor is represented by vdw surface (white). Cholesterol is represented by white stick (red stick for the O atoms) and the vdw surface (yellow and transparent), inset: Residues lining up the hydrophobic face of the H8 (Q821, K823, V824, V825, S826, R829, and A830). (**C**) The distribution of the O atoms of the OH group of the cholesterol in a shell of 2 Å around the mGluR2 receptor (TM1-TM7-H8 new cartoon and purple). In this case we concatenated all the MD runs of the simulations with cholesterol and we sampled the O location every 10 ns. The O atoms (dot colored) are represented according to the time scale evolution using a red-white-blue time scale, in which red represents the early location of the O atoms while blue the final one. Highlighted in the black box a membrane view of the O location.
(DOCX)

Figure S3 **Typical cholesterol contacts with mGluR2.** Representation of the direct cholesterol contacts with the mGluR2-H8, as example we reported the typical interaction maps among mGluR2 and cholesterols (**A, B**) and the typical profile of the molecular surface representation of the cholesterol pocket (**C**). Two cholesterol molecules bind a cleft described by TM1/TM7 and H8. **(A)** And **(B)** represent maps of the interaction among the cholesterol molecules and the mGluR2 receptor. (**C**) Surface representation of the pocket hosting the cholesterol molecules, in green the hydrophobic region, in purple the polar region. It can be appreciate how the OH group of the cholesterol molecules point towards the polar portion of the H8 represented by the R829 residue and the backbone portion of the S827, and A830.
(DOCX)

Figure S4 **Probability Density Functions (PDF) of the collected simulations with cholesterol.** PDF plots of the 10 MD runs with cholesterol, with different sampling methods (**A–B**). (**C**) PDF plot of a single MD run.
(DOCX)

Figure S5 **Probability Density Functions (PDF) of the collected simulations without cholesterol.** PDF plots of the 10 MD runs without cholesterol, with different sampling methods (**A–B**). (**C**) PDF plot of a single MD run in which the misfolding occurs; and (**D**) PDF plot of a single MD run in which the misfolding event does not occur.
(DOCX)

Figure S6 **EDP of the membrane bilayer.** Average EDP of the whole bilayer (A) and EDP of the PO_4_ groups (B). In blue the average EDP for the simulation with cholesterol, while the simulation without cholesterol is the brown one.
(DOCX)

Figure S7 **Prediction of the amphipathic character of different H8 sequences.** (**A**) Amphipathic character and (**B**) hydrophobic moment of the residues forming the H8 sequences of each receptor. The blue histograms represent the amphipathic (**A**) and the hydrophobic moment (**B**) values. The black lines are the regression lines, while the red curve described the polynomial tendency for the amphipathic and hydrophobic moment values respectively.
(DOCX)

Figure S8 **Prediction of the amphipathic character of the mGluR-8.** (**A**) Amphipathic character and (**B**) hydrophobic moment of the residues forming the H8 sequences of β_1–2_ (b1–2), D_2_ (d2) and A_2A_ (A2A) receptors. The blue histograms represent the amphipathic (**A**) and the hydrophobic moment (**B**) values. The black lines are the regression lines, while the red curve described the polynomial tendency for the amphipathic and hydrophobic moment values respectively.
(DOCX)

Figure S9 **Human mGluR2-bovine rhodopsin alignment.** Highlighted in red the TM regions, in green the super-conserved residues for class A GPCRs on each TM, in yellow the residues forming the allosteric binding pocket for the RO4988546 and RO5488608 compounds.
(DOCX)

Figure S10 **Alignment comparison.** Comparison of the new **(A)** and the old **(B)** alignment for the TM5.
(DOCX)

Figure S11 **Analysis of the structural properties of the generated mGluR2 model. (A)** Disulphide bond between the residues of C^3^.^25^ and C^EL2^.^50^; **(B)** ionic interactions at the bottom part (intracellular end of the TM domains) of the mGluR2 receptor.
(DOCX)

Figure S12 **Analysis of the docking studies.** Representation of the docking pose for the RO4988546 (**A**) and RO5488608 (**B**) compounds and the ligand-receptor interaction C and D respectively.
(DOCX)

Figure S13 **EDP scheme.** At each simulation frame**,** the EDP is computed by summing the atomic number (Z) and the partial charges of the atoms falling into 1 A-thick slabs parallel to the z axis. The sum, normalized by volume, provides the local 1-D density value around z.
(DOCX)

Table S1 **Percentage of conservation of the helix content for the mGluR2-H8.** Conservation of the H8 structure in the last frame of each simulation expressed as % of H-bonds formed by backbone atoms which stabilize the α-helical structures
(DOCX)

Table S2 **Structural details of the generated receptor-membrane complexes.** Final structural properties of the membrane-receptor complex.
(DOCX)

Table S3 **MD parameter settings.** Parameters used for the MD equilibration and production phases.
(DOCX)

Table S4 **Cut-off used for the cluster analysis.**
(DOCX)

Chart S1 **Representation of the 2D structures of the compounds used for the docking studies.** Compounds used for the docking studies on the mGluR2 receptor. RO4988546 (A) and RO5488608 (B).
(DOCX)

Experimental Procedures S1 **Detailed description of the material and methods employed in the work.** Generation/validation of the mGluR2-membrane model, MD protocols, calculation of PDF and ED plots, and structural prediction protocol.
(DOC)

Movie S1 **Dynamic properties of the mGluR2-H8 domain in the presence and absence of cholesterol.** Movie comparing the dynamic properties of the amphipathic H8 in cholesterol-rich (left) and -depleted (right) systems.
(M4V)
