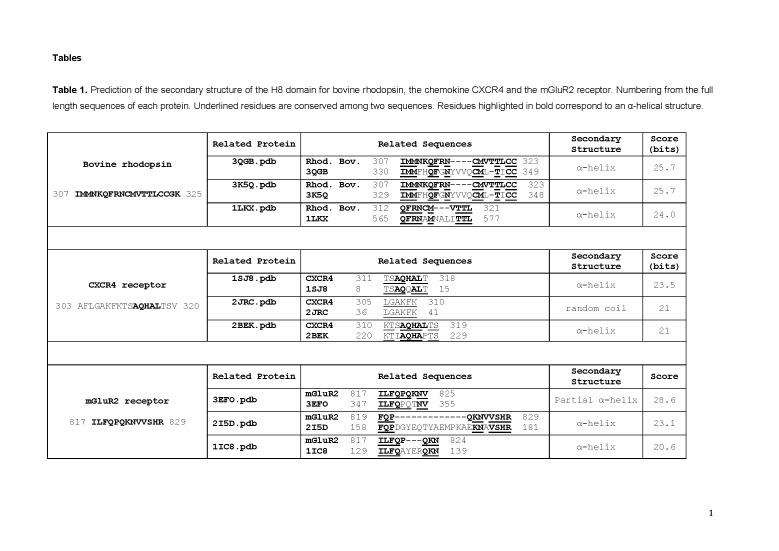# Correction: Membrane-Sensitive Conformational States of Helix 8 in the Metabotropic Glu2 Receptor, a Class C GPCR

**DOI:** 10.1371/annotation/b3d4540a-9b4b-4855-b570-6324b40232fe

**Published:** 2012-11-14

**Authors:** Agostino Bruno, Gabriele Costantino, Gianni de Fabritiis, Manuel Pastor, Jana Selent

There were formatting errors in Table 1. The correct Table 1 can be viewed here: 

**Figure:**