# Supplementary material for: Membrane-Sensitive Conformational States of Helix 8 in the Metabotropic Glu2 Receptor, a Class C GPCR
Source: PLoS One. 2012 Aug 1;7(8):e42023. doi: 10.1371/journal.pone.0042023 (PMC3411606; doi:10.1371/journal.pone.0042023)
Supplement: Figure S1 — Typical average RMSD of the mGluR2. (A) Average RMSD per residue of the Cα atoms of the mGluR2 receptor. The blue line refers to the simulation with cholesterol (25%), the red one refers to the simulation without cholesterol (0%). The regions highlighted in grey represent the transmembrane regions (TM1–7) and the Helix 8 (H8). (B) RMSD of Cα atoms of mGluR2 with (blue) and without (brown) cholesterol for a single MD run. (DOCX) [file pone.0042023.s001.docx]

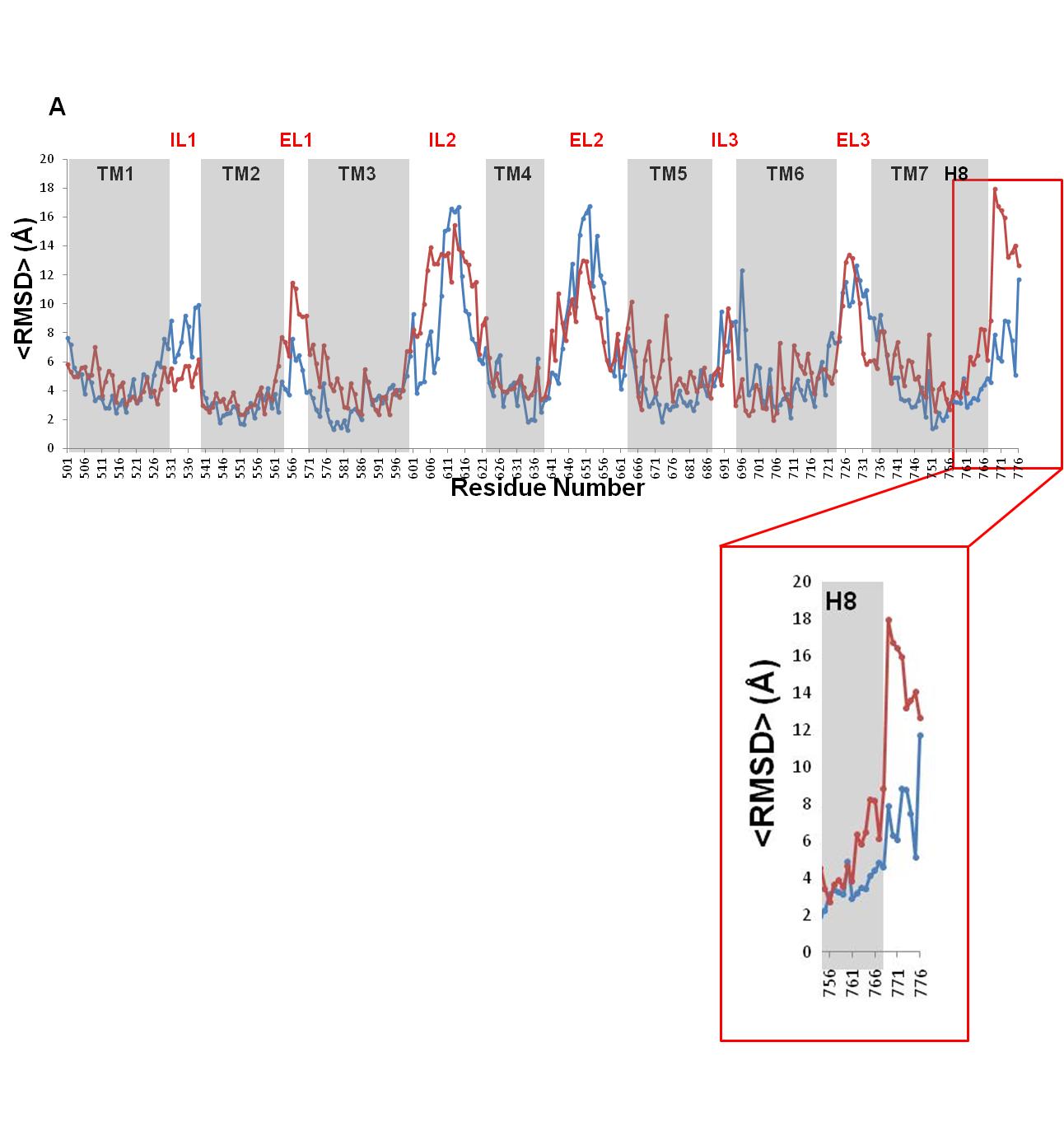


**
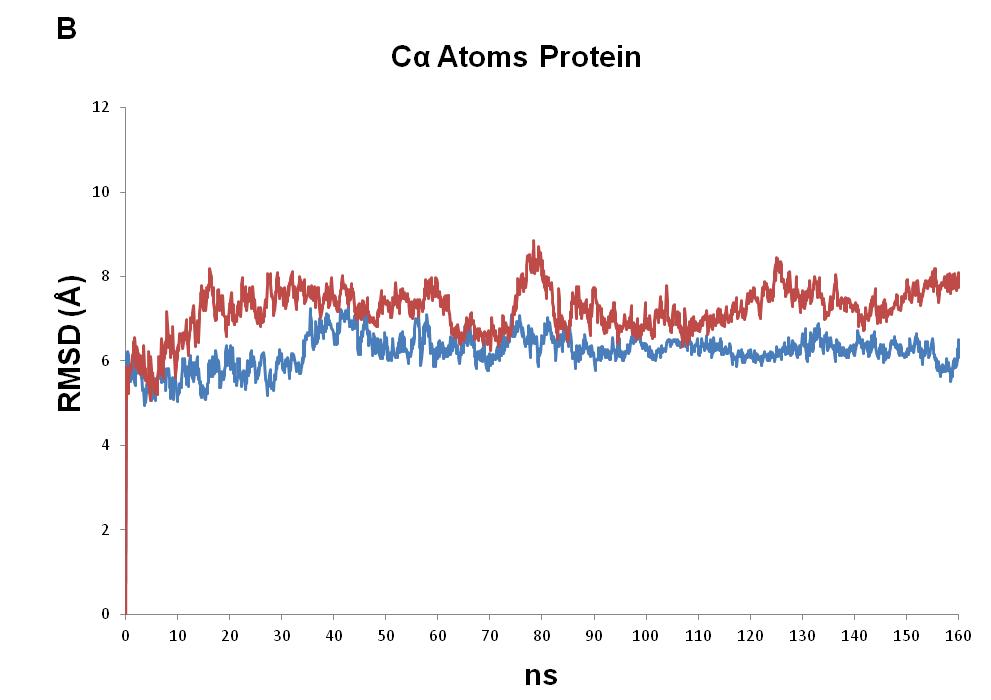
**

**Figure S1. Typical average RMSD of the mGluR2. (A)** Average RMSD per residue of the Cα atoms of the mGluR2 receptor. The blue line refers to the simulation with cholesterol (25%), the red one refers to the simulation without cholesterol (0%). The regions highlighted in grey represent the transmembrane regions (TM1-7) and the Helix 8 (H8). (**B**) RMSD of Cα atoms of mGluR2 with (blue) and without (brown) cholesterol for a single MD run.
