# Supplementary material for: Membrane-Sensitive Conformational States of Helix 8 in the Metabotropic Glu2 Receptor, a Class C GPCR
Source: PLoS One. 2012 Aug 1;7(8):e42023. doi: 10.1371/journal.pone.0042023 (PMC3411606; doi:10.1371/journal.pone.0042023)
Supplement: Figure S2 — Representation of the putative cholesterol pocket. Representation of direct cholesterol contacts with the mGluR2 receptor. (A–B) Different views of the cholesterol cleft described by TM1-TM7-H8 (new cartoon and purple, and transparent and white) the whole receptor is represented by vdw surface (white). Cholesterol is represented by white stick (red stick for the O atoms) and the vdw surface (yellow and transparent), inset: Residues lining up the hydrophobic face of the H8 (Q821, K823, V824, V825, S826, R829, and A830). (C) The distribution of the O atoms of the OH group of the cholesterol in a shell of 2 Å around the mGluR2 receptor (TM1-TM7-H8 new cartoon and purple). In this case we concatenated all the MD runs of the simulations with cholesterol and we sampled the O location every 10 ns. The O atoms (dot colored) are represented according to the time scale evolution using a red-white-blue time scale, in which red represents the early location of the O atoms while blue the final one. Highlighted in the black box a membrane view of the O location. (DOCX) [file pone.0042023.s002.docx]

**
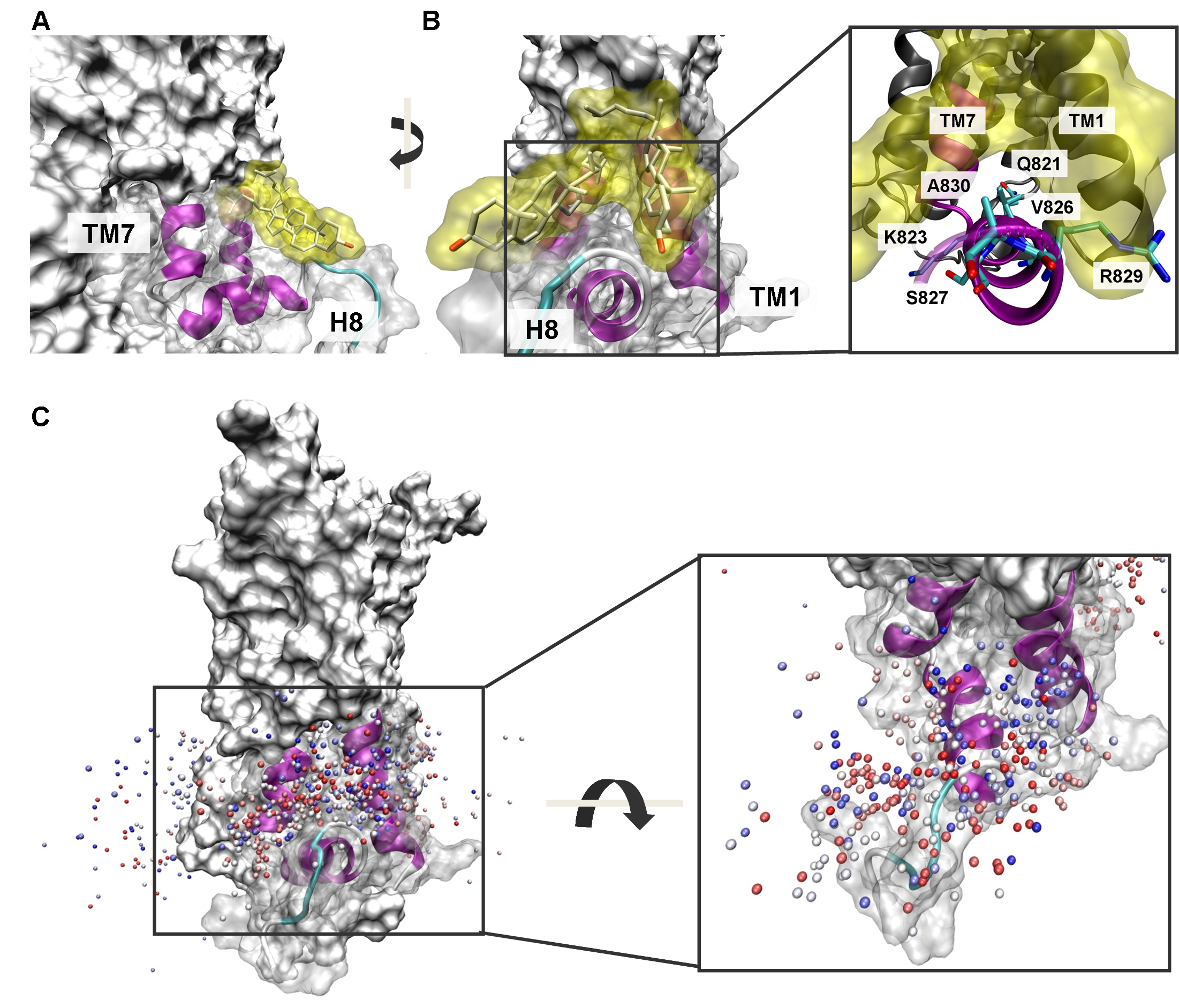
**

**Figure S2. Representation of the putative cholesterol pocket.** Representation of direct cholesterol contacts with the mGluR2 receptor. (**A-B**) Different views of the cholesterol cleft described by TM1-TM7-H8 (new cartoon and purple, and transparent and white) the whole receptor is represented by vdw surface (white). Cholesterol is represented by white stick (red stick for the O atoms) and the vdw surface (yellow and transparent), inset: Residues lining up the hydrophobic face of the H8 (Q821, K823, V824, V825, S826, R829, and A830). (**C**) The distribution of the O atoms of the OH group of the cholesterol in a shell of 2 Å around the mGluR2 receptor (TM1-TM7-H8 new cartoon and purple). In this case we concatenated all the MD runs of the simulations with cholesterol and we sampled the O location every 10 ns. The O atoms (dot colored) are represented according to the time scale evolution using a red-white-blue time scale, in which red represents the early location of the O atoms while blue the final one. Highlighted in the black box a membrane view of the O location.
