# Supplementary material for: Membrane-Sensitive Conformational States of Helix 8 in the Metabotropic Glu2 Receptor, a Class C GPCR
Source: PLoS One. 2012 Aug 1;7(8):e42023. doi: 10.1371/journal.pone.0042023 (PMC3411606; doi:10.1371/journal.pone.0042023)
Supplement: Figure S3 — Typical cholesterol contacts with mGluR2. Representation of the direct cholesterol contacts with the mGluR2-H8, as example we reported the typical interaction maps among mGluR2 and cholesterols (A, B) and the typical profile of the molecular surface representation of the cholesterol pocket (C). Two cholesterol molecules bind a cleft described by TM1/TM7 and H8. (A) And (B) represent maps of the interaction among the cholesterol molecules and the mGluR2 receptor. (C) Surface representation of the pocket hosting the cholesterol molecules, in green the hydrophobic region, in purple the polar region. It can be appreciate how the OH group of the cholesterol molecules point towards the polar portion of the H8 represented by the R829 residue and the backbone portion of the S827, and A830. (DOCX) [file pone.0042023.s003.docx]

**
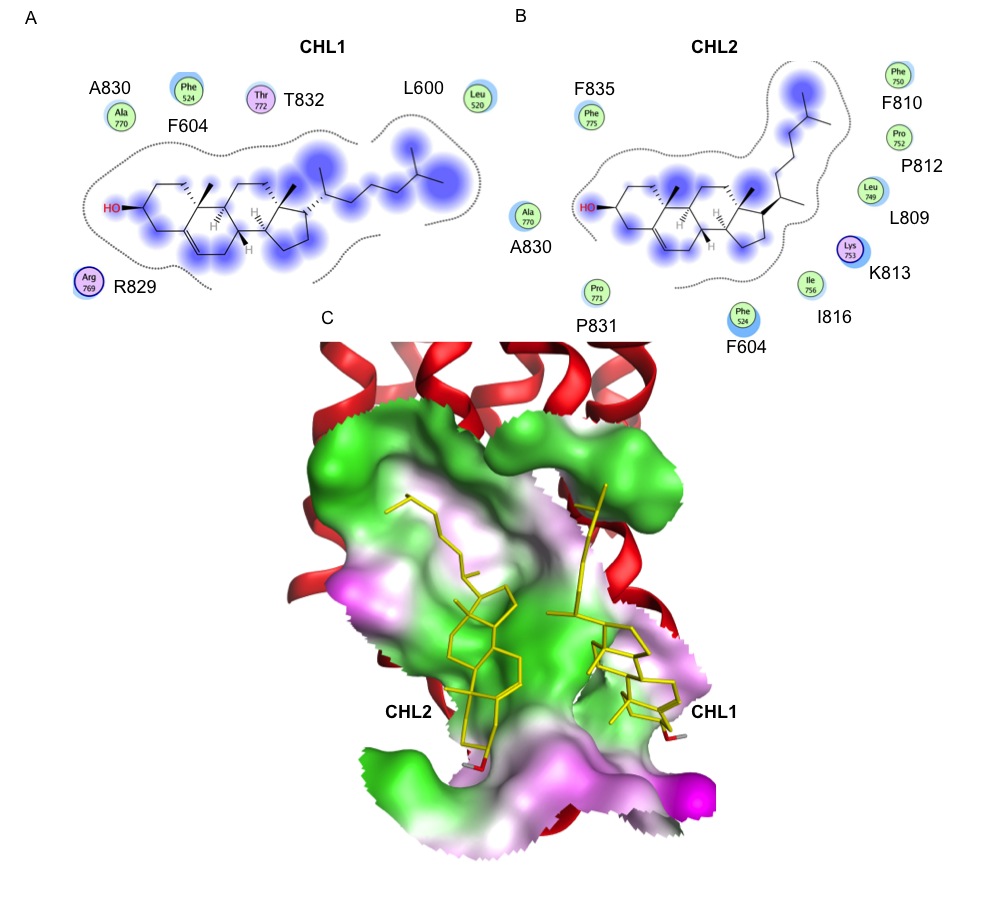
**

**Figure S3. Typical cholesterol contacts with mGluR2.** Representation of the direct cholesterol contacts with the mGluR2-H8, as example we reported the typical interaction maps among mGluR2 and cholesterols (**A, B**) and the typical profile of the molecular surface representation of the cholesterol pocket (**C**). Two cholesterol molecules bind a cleft described by TM1/TM7 and H8. **(A)** And **(B)** represent maps of the interaction among the cholesterol molecules and the mGluR2 receptor. (**C**) Surface representation of the pocket hosting the cholesterol molecules, in green the hydrophobic region, in purple the polar region. It can be appreciate how the OH group of the cholesterol molecules point towards the polar portion of the H8 represented by the R829 residue and the backbone portion of the S827, and A830.
