# Supplementary material for: Membrane-Sensitive Conformational States of Helix 8 in the Metabotropic Glu2 Receptor, a Class C GPCR
Source: PLoS One. 2012 Aug 1;7(8):e42023. doi: 10.1371/journal.pone.0042023 (PMC3411606; doi:10.1371/journal.pone.0042023)
Supplement: Figure S5 — Probability Density Functions (PDF) of the collected simulations without cholesterol. PDF plots of the 10 MD runs without cholesterol, with different sampling methods (A–B). (C) PDF plot of a single MD run in which the misfolding occurs; and (D) PDF plot of a single MD run in which the misfolding event does not occur. (DOCX) [file pone.0042023.s005.docx]

**
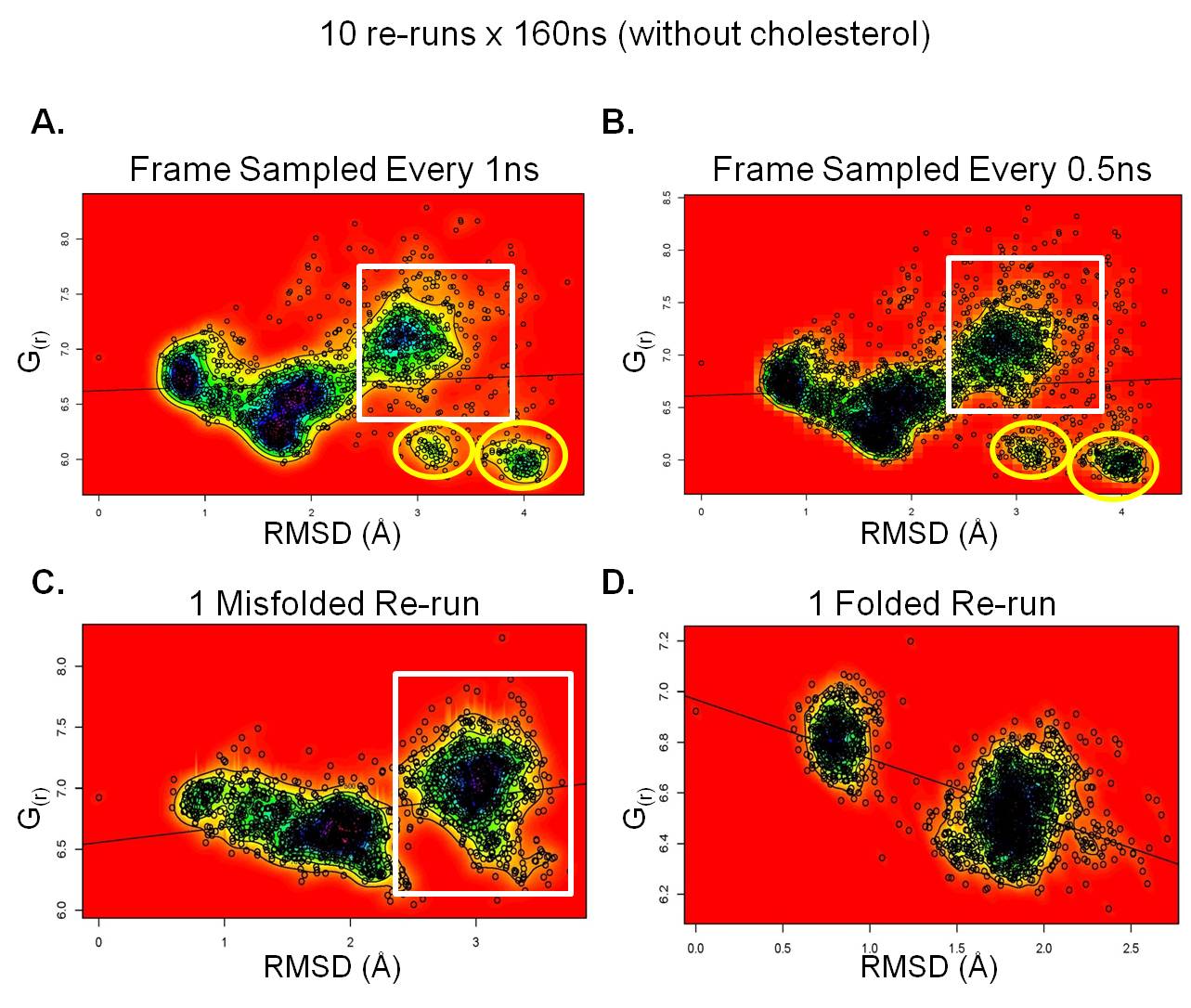
**

**Figure S5. Probability Density Functions (PDF) of the collected simulations without cholesterol.** PDF plots of the 10 MD runs without cholesterol, with different sampling methods (**A-B**). (**C**) PDF plot of a single MD run in which the misfolding occurs; and (**D**) PDF plot of a single MD run in which the misfolding event does not occur.
