# Supplementary material for: Membrane-Sensitive Conformational States of Helix 8 in the Metabotropic Glu2 Receptor, a Class C GPCR
Source: PLoS One. 2012 Aug 1;7(8):e42023. doi: 10.1371/journal.pone.0042023 (PMC3411606; doi:10.1371/journal.pone.0042023)
Supplement: Figure S6 — EDP of the membrane bilayer. Average EDP of the whole bilayer (A) and EDP of the PO4 groups (B). In blue the average EDP for the simulation with cholesterol, while the simulation without cholesterol is the brown one. (DOCX) [file pone.0042023.s006.docx]

**
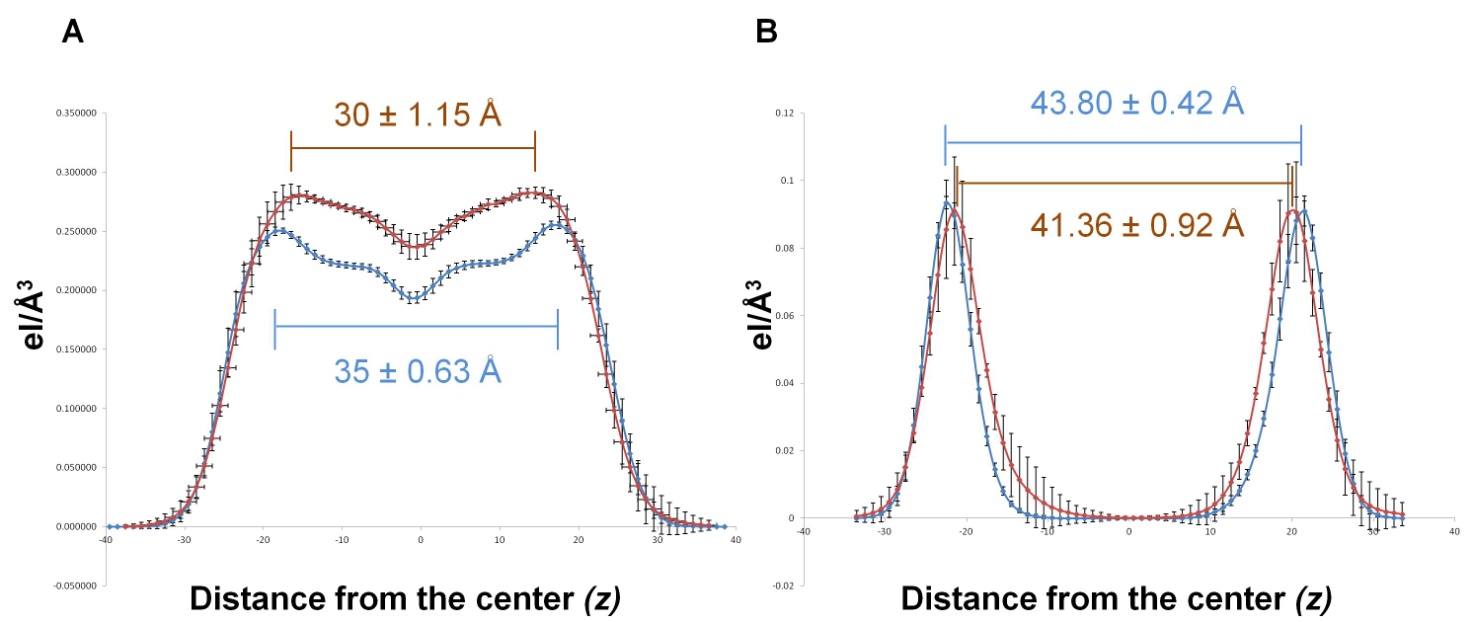
**

**Figure S6.** **EDP of the membrane bilayer.** Average EDP of the whole bilayer (A) and EDP of the PO_4_ groups (B). In blue the average EDP for the simulation with cholesterol, while the simulation without cholesterol is the brown one.
