# Supplementary material for: Membrane-Sensitive Conformational States of Helix 8 in the Metabotropic Glu2 Receptor, a Class C GPCR
Source: PLoS One. 2012 Aug 1;7(8):e42023. doi: 10.1371/journal.pone.0042023 (PMC3411606; doi:10.1371/journal.pone.0042023)
Supplement: Figure S7 — Prediction of the amphipathic character of different H8 sequences. (A) Amphipathic character and (B) hydrophobic moment of the residues forming the H8 sequences of each receptor. The blue histograms represent the amphipathic (A) and the hydrophobic moment (B) values. The black lines are the regression lines, while the red curve described the polynomial tendency for the amphipathic and hydrophobic moment values respectively. (DOCX) [file pone.0042023.s007.docx]

**
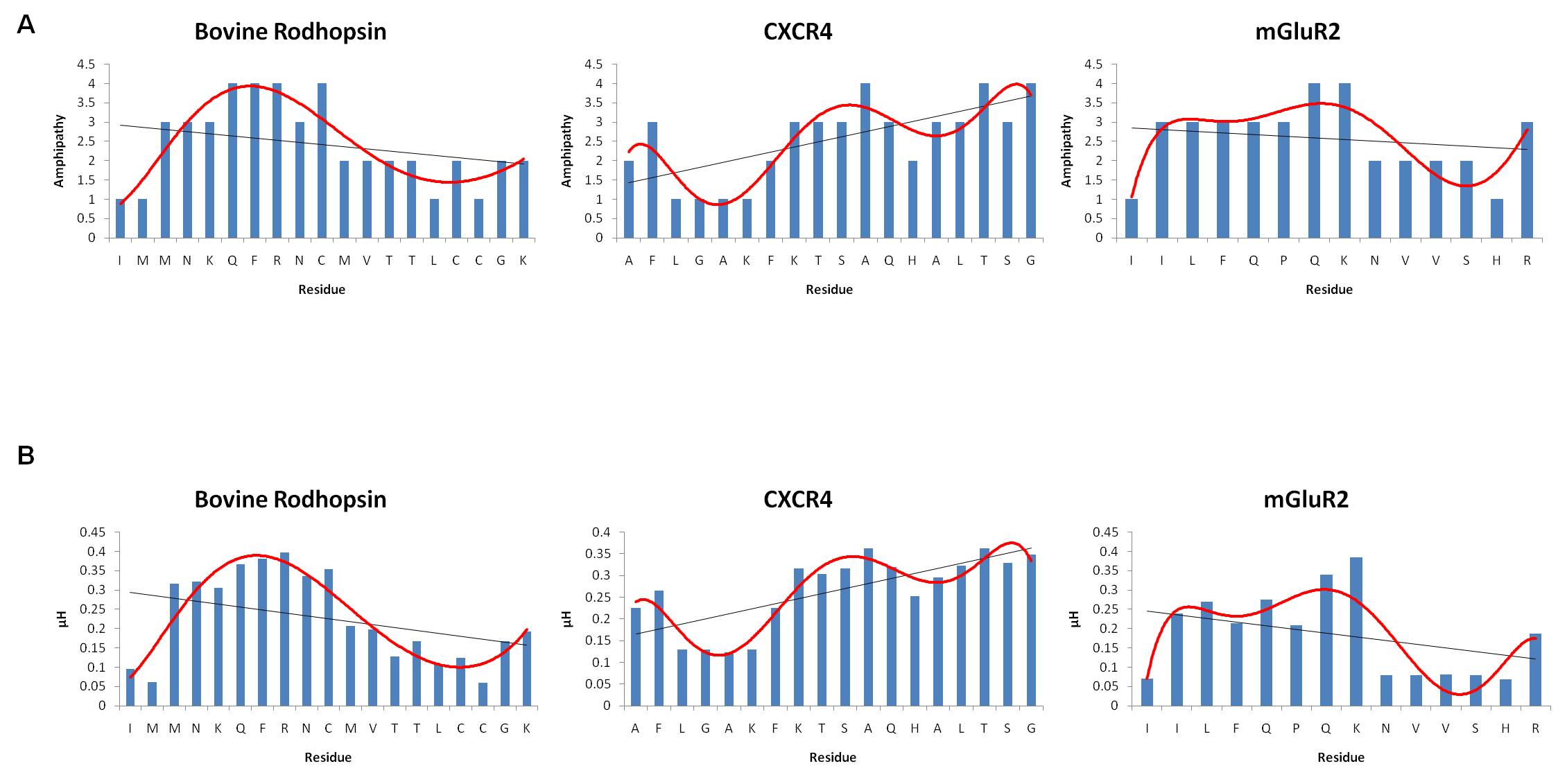
**

**Figure S7. Prediction of the amphipathic character of different H8 sequences.** (**A**) Amphipathic character and (**B**) hydrophobic moment of the residues forming the H8 sequences of each receptor. The blue histograms represent the amphipathic (**A**) and the hydrophobic moment (**B**) values. The black lines are the regression lines, while the red curve described the polynomial tendency for the amphipathic and hydrophobic moment values respectively.
