# Supplementary material for: Membrane-Sensitive Conformational States of Helix 8 in the Metabotropic Glu2 Receptor, a Class C GPCR
Source: PLoS One. 2012 Aug 1;7(8):e42023. doi: 10.1371/journal.pone.0042023 (PMC3411606; doi:10.1371/journal.pone.0042023)
Supplement: Figure S9 — Human mGluR2-bovine rhodopsin alignment. Highlighted in red the TM regions, in green the super-conserved residues for class A GPCRs on each TM, in yellow the residues forming the allosteric binding pocket for the RO4988546 and RO5488608 compounds. (DOCX) [file pone.0042023.s009.docx]

sp|P02699|OPSD_BOVIN MNGTEGPNFYVPFSNKTGVVRSPFEAPQYYLAEPWQFSMLAAYMFLLIMLGFPINFLTLY

sp|Q14416|GRM2_HUMAN ---------------------------------IRWGDAWAVGPVTIACLGALATLFVLG

sp|P02699|OPSD_BOVIN VTVQHKKLRT--PLNYILLNLAVADLFMVFGGFTTTLYTSLHGYFVFGPTGCNLEGFFAT

sp|Q14416|GRM2_HUMAN VFVRHNATPVVKASGRELCYILLGGVFLCYCMTFIFIA----KPST---AVCTLRRLGLG

sp|P02699|OPSD_BOVIN LGGEIALWSLVVLAIERYVVVCKPMSNFRFG----------ENHAIMGVAFTWVMALACA

sp|Q14416|GRM2_HUMAN TAFSVCYSALLTKTNRIARIFGGAREGAQRPRFISPASQVAICLALISGQLLIVVAWLVV

sp|P02699|OPSD_BOVIN APPLVGWSRYIPEGMQ------CSCGIDYYTPHEETNNESFVIYMFVVHFIIPLIVIFFC

sp|Q14416|GRM2_HUMAN EAPGTGKE----TAPERREVVTLRCNHR----------DASMLGSLAYNVLLIALCTLYA

sp|P02699|OPSD_BOVIN YGQLVFTV-KEAAAQQQEATTQKAEKEVTRMVIIMVIAFLICWLPYAGVAFYIFTHQGSD

sp|Q14416|GRM2_HUMAN FKTRK----------CPENF------NEAKFIGFTMYTTCIIWLAFLPIFYVTSSDYRVQ

sp|P02699|OPSD_BOVIN FGPIF-MTIPAFFAKTSAVYNPVIYIMMNKQFRNCMVTTLCCGKNPLGDDEASTTVSKTE

sp|Q14416|GRM2_HUMAN TTTMCVSVSLSGSVVLGCLFAPKLHIILFQPQKNVVSHRAPT--SRFGSAAARASSSLGQ

sp|P02699|OPSD_BOVIN TSQVAPA

sp|Q14416|GRM2_HUMAN GSG----

**Figure S9. Human mGluR2-bovine rhodopsin alignment.** Highlighted in red the TM regions, in green the super-conserved residues for class A GPCRs on each TM, in yellow the residues forming the allosteric binding pocket for the RO4988546 and RO5488608 compounds.
