# Supplementary figures and images for: Membrane-Sensitive Conformational States of Helix 8 in the Metabotropic Glu2 Receptor, a Class C GPCR
Source: PLoS One. 2012 Aug 1;7(8):e42023. doi: 10.1371/journal.pone.0042023 (PMC3411606; doi:10.1371/journal.pone.0042023)

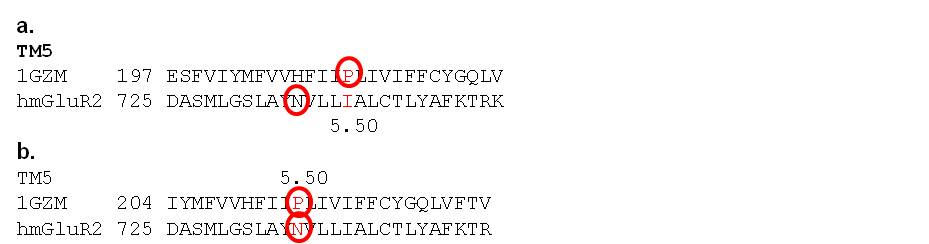


**Figure S10. Alignment comparison.** Comparison of the new **(A)** and the old **(B)** alignment for the TM5.

Supplement: Figure S10 — Alignment comparison. Comparison of the new (A) and the old (B) alignment for the TM5. (DOCX) [file pone.0042023.s010.docx]
