# Supplementary material for: Membrane-Sensitive Conformational States of Helix 8 in the Metabotropic Glu2 Receptor, a Class C GPCR
Source: PLoS One. 2012 Aug 1;7(8):e42023. doi: 10.1371/journal.pone.0042023 (PMC3411606; doi:10.1371/journal.pone.0042023)
Supplement: Figure S11 — Analysis of the structural properties of the generated mGluR2 model. (A) Disulphide bond between the residues of C3.25 and CEL2.50; (B) ionic interactions at the bottom part (intracellular end of the TM domains) of the mGluR2 receptor. (DOCX) [file pone.0042023.s011.docx]

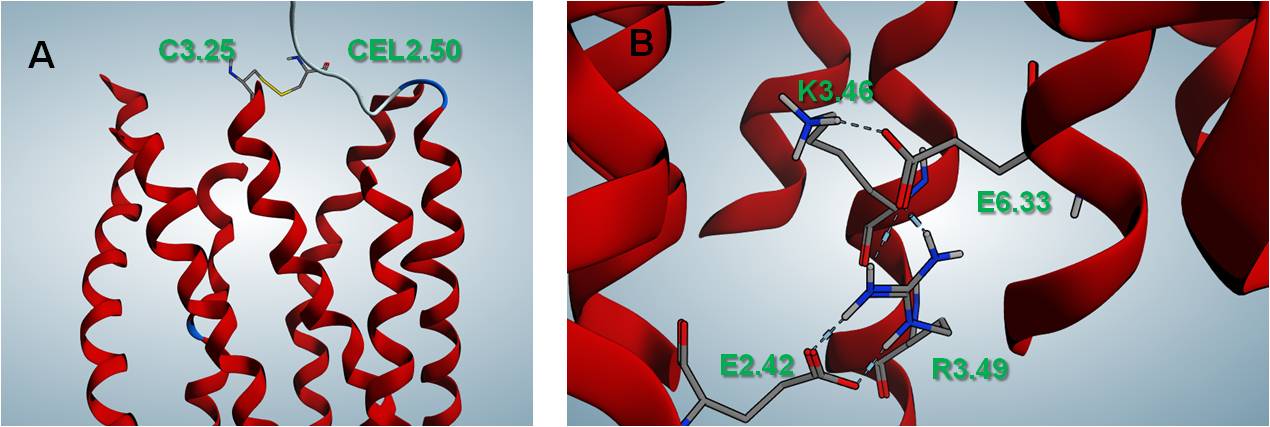


**Figure S11. Analysis of the structural properties of the generated mGluR2 model. (A)** Disulphide bond between the residues of C^3.25^ and C^EL2.50^; **(B)** ionic interactions at the bottom part (intracellular end of the TM domains) of the mGluR2 receptor.
