# Supplementary material for: Membrane-Sensitive Conformational States of Helix 8 in the Metabotropic Glu2 Receptor, a Class C GPCR
Source: PLoS One. 2012 Aug 1;7(8):e42023. doi: 10.1371/journal.pone.0042023 (PMC3411606; doi:10.1371/journal.pone.0042023)
Supplement: Figure S12 — Analysis of the docking studies. Representation of the docking pose for the RO4988546 (A) and RO5488608 (B) compounds and the ligand-receptor interaction C and D respectively. (DOCX) [file pone.0042023.s012.docx]

**
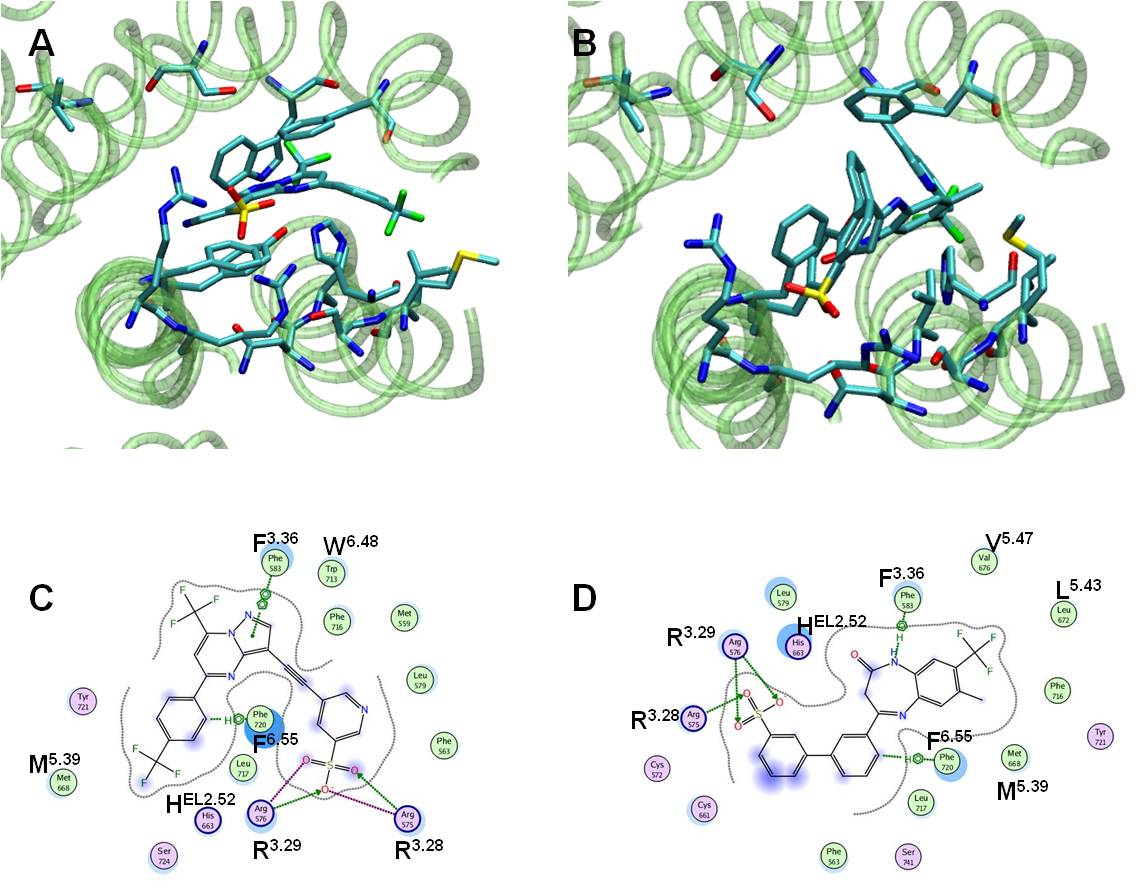
**

**Figure S12. Analysis of the docking studies.** Representation of the docking pose for the RO4988546 (**A**) and RO5488608 (**B**) compounds and the ligand-receptor interaction C and D respectively.
