# Supplementary material for: Membrane-Sensitive Conformational States of Helix 8 in the Metabotropic Glu2 Receptor, a Class C GPCR
Source: PLoS One. 2012 Aug 1;7(8):e42023. doi: 10.1371/journal.pone.0042023 (PMC3411606; doi:10.1371/journal.pone.0042023)
Supplement: Figure S13 — EDP scheme. At each simulation frame, the EDP is computed by summing the atomic number (Z) and the partial charges of the atoms falling into 1 A-thick slabs parallel to the z axis. The sum, normalized by volume, provides the local 1-D density value around z. (DOCX) [file pone.0042023.s013.docx]

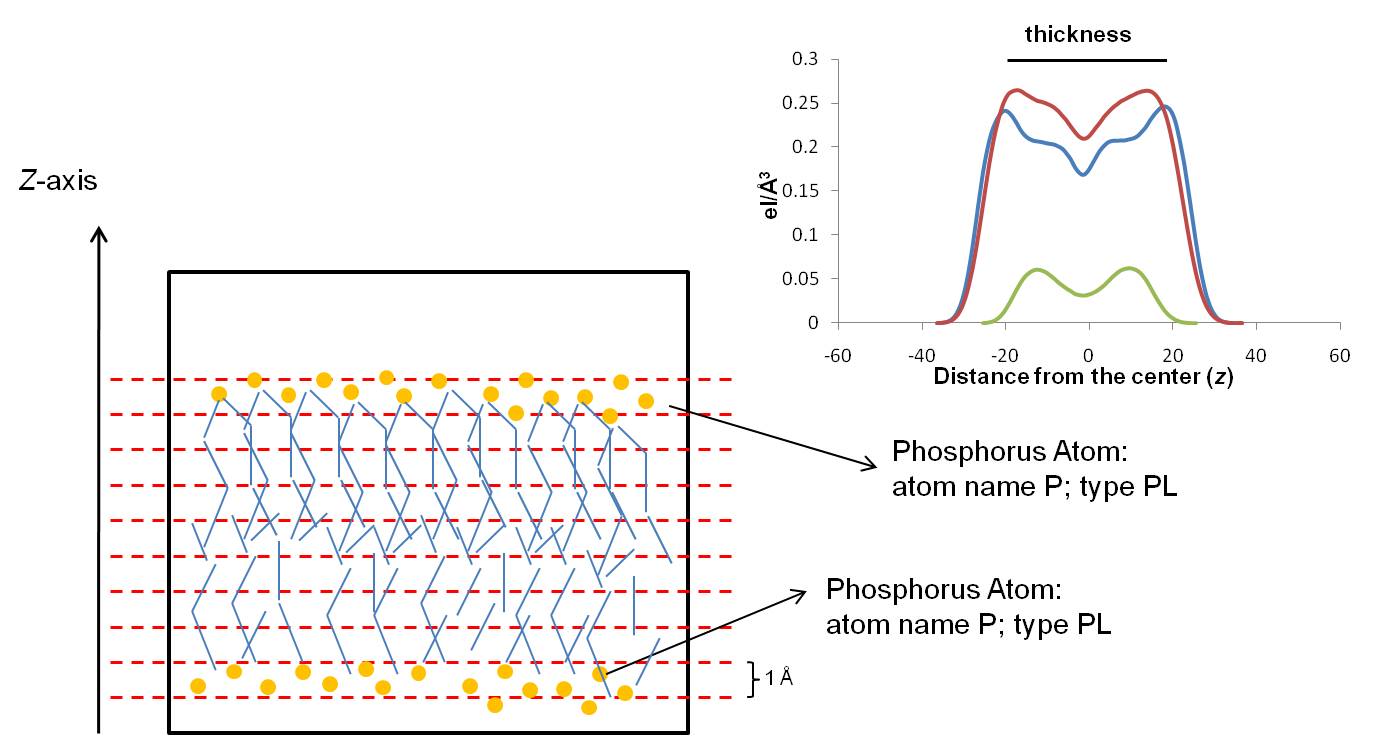


**Figure S13. EDP scheme.** At each simulation frame**,** the EDP is computed by summing the atomic number (Z) and the partial charges of the atoms falling into 1 A-thick slabs parallel to the z axis. The sum, normalized by volume, provides the local 1-D density value around z.
