# Supplementary material for: Membrane-Sensitive Conformational States of Helix 8 in the Metabotropic Glu2 Receptor, a Class C GPCR
Source: PLoS One. 2012 Aug 1;7(8):e42023. doi: 10.1371/journal.pone.0042023 (PMC3411606; doi:10.1371/journal.pone.0042023)
Supplement: Table S1 — Percentage of conservation of the helix content for the mGluR2-H8. Conservation of the H8 structure in the last frame of each simulation expressed as % of H-bonds formed by backbone atoms which stabilize the α-helical structures (DOCX) [file pone.0042023.s014.docx]

**Table S1. Percentage of conservation of the helix content for the mGluR2-H8.** Conservation of the H8 structure in the last frame of each simulation expressed as % of H-bonds formed by backbone atoms which stabilize the α-helical structures

| **Runs** | **α-helical H8 structure [%]** | |
| --- | --- | --- |
|  | **cholesterol-depleted** | **cholesterol-rich** |
| **run 1** | 30 | 100 |
| **run 2** | 0 | 100 |
| **run 3** | 20 | 100 |
| **run 4** | 100 | 100 |
| **run 5** | 100 | 100 |
| **run 6** | 100 | 100 |
| **run 7** | 100 | 100 |
| **run 8** | 20 | 100 |
| **run 9** | 30 | 100 |
| **run 10** | 40 | 100 |
| **stability** | **60% unstable** | **0% unstable** |
