# Supplementary material for: Membrane-Sensitive Conformational States of Helix 8 in the Metabotropic Glu2 Receptor, a Class C GPCR
Source: PLoS One. 2012 Aug 1;7(8):e42023. doi: 10.1371/journal.pone.0042023 (PMC3411606; doi:10.1371/journal.pone.0042023)
Supplement: Table S2 — Structural details of the generated receptor-membrane complexes. Final structural properties of the membrane-receptor complex. (DOCX) [file pone.0042023.s015.docx]

**Table S2. Structural details of the generated receptor-membrane complexes.** Final structural properties of the membrane-receptor complex.

| **Membrane-Receptor Complex** | | | | | | | |
| --- | --- | --- | --- | --- | --- | --- | --- |
| **mGluR2** | | | | | | | |
| **0%**  **Chol** | | | | **25%**  **Chol** | | | |
| **System Size**  **(Å)** | | | | **System Size**  **(Å)** | | | |
| ***X*** | ***Y*** | | ***z*** | ***x*** | ***y*** | | ***z*** |
| 94 | 94 | | 110 | 94 | 94 | | 110 |
| **Number of Lipids** | | | | **Number of Lipids** | | | |
| 200 SDPC | | | | 60 Chol  /  180 SDPC | | | |
| **Ions** | | | | **Ions** | | | |
| **SOD** | | **CLA** | | **SOD** | | **CLA** | |
| 44 | | 60 | | 44 | | 60 | |
| **Waters** | | | | **Waters** | | | |
| 18231 | | | | 18282 | | | |
| **Box Type** | | | | **Box Type** | | | |
| Rectangle | | | | Rectangle | | | |
