# Supplementary material for: Membrane-Sensitive Conformational States of Helix 8 in the Metabotropic Glu2 Receptor, a Class C GPCR
Source: PLoS One. 2012 Aug 1;7(8):e42023. doi: 10.1371/journal.pone.0042023 (PMC3411606; doi:10.1371/journal.pone.0042023)
Supplement: Table S3 — MD parameter settings. Parameters used for the MD equilibration and production phases. (DOCX) [file pone.0042023.s016.docx]

**Table S3. MD parameter settings.** Parameters used for the MD equilibration and production phases.

| **Relaxation Phase** | | | | | | | | | |
| --- | --- | --- | --- | --- | --- | --- | --- | --- | --- |
|  | **Timestep** | **Numstep** | **Temperature** | **Harmonic Constraints**  **(kcal/(mol*Å^2^))** | | | | | **Constant Temperature control** |
|  | | | | ***Backbone*** | ***Sidechains*** | ***Lipids*** | ***Waters*** | ***Ions*** |  |
| ***Step1*** | 2.0 fs | 10 ns | 200 K | 10 | 10 | - | - | - | NPT |
| ***Step2*** | 2.0 fs | 10 ns | 300 K | - | - | - | - | - | NPT |
| **Production Phase** | | | | | | | | | |
| ***Step1*** | 4.0 fs | 160 ns | 300 K | **-** | **-** | **-** | **-** | **-** | NPT |
