# Supplementary material for: Membrane-Sensitive Conformational States of Helix 8 in the Metabotropic Glu2 Receptor, a Class C GPCR
Source: PLoS One. 2012 Aug 1;7(8):e42023. doi: 10.1371/journal.pone.0042023 (PMC3411606; doi:10.1371/journal.pone.0042023)
Supplement: Table S4 — Cut-off used for the cluster analysis. (DOCX) [file pone.0042023.s017.docx]

**Table S4. Cut-off used for the cluster analysis.**

**A.**

| **Cluster** | **RMSD**  **cut off** | **G_(r)_**  **cut off** |
| --- | --- | --- |
| **1** | <1 | > 6.5 |
| **2** | 1.2-2.2 | < 6.8 |
| **3** | 2.7-3.6 | > 6.8 |
| **4** | 3-3.5 | < 6.4 |
| **5** | > 3.5 | < 6.1 |

**B.**

| **Cluster** | **RMSD**  **cut off** | **G_(r)_**  **cut off** |
| --- | --- | --- |
| **1** | 0.5-1.5 | 6.5-7.4 |
| **2** | 1.5-2.5 | 6.3-7.0 |
