# Supplementary material for: Membrane-Sensitive Conformational States of Helix 8 in the Metabotropic Glu2 Receptor, a Class C GPCR
Source: PLoS One. 2012 Aug 1;7(8):e42023. doi: 10.1371/journal.pone.0042023 (PMC3411606; doi:10.1371/journal.pone.0042023)
Supplement: Chart S1 — Representation of the 2D structures of the compounds used for the docking studies. Compounds used for the docking studies on the mGluR2 receptor. RO4988546 (A) and RO5488608 (B). (DOCX) [file pone.0042023.s018.docx]

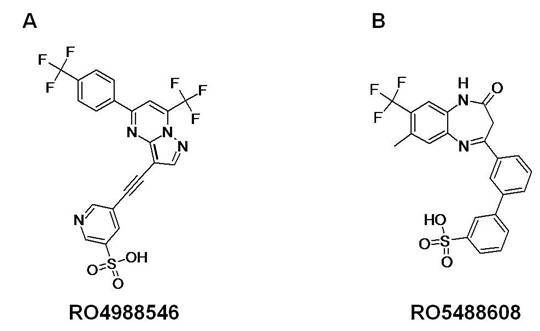


**Chart S1. Representation of the 2D structures of the compounds used for the docking studies.** Compounds used for the docking studies on the mGluR2 receptor. RO4988546 (**A**) and RO5488608 (**B**).
