# Supplementary material for: Membrane-Sensitive Conformational States of Helix 8 in the Metabotropic Glu2 Receptor, a Class C GPCR
Source: PLoS One. 2012 Aug 1;7(8):e42023. doi: 10.1371/journal.pone.0042023 (PMC3411606; doi:10.1371/journal.pone.0042023)
Supplement: Experimental Procedures S1 — Detailed description of the material and methods employed in the work. Generation/validation of the mGluR2-membrane model, MD protocols, calculation of PDF and ED plots, and structural prediction protocol. (DOC) [file pone.0042023.s019.doc]

**Supplemental Information**

Membrane-Sensitive Conformational States of Helix 8 in the Metabotropic Glu2 Receptor, a Class C GPCR

Agostino Bruno^a,b^, Gabriele Costantino^b*^, Gianni de Fabritiis^c^, Manuel Pastor^a^ and Jana Selent^a^*.

*^a^Computer-Assisted Drug Design Lab, Research Unit on Biomedical Informatics (GRIB), Universitat Pompeu Fabra, Dr Aiguader 88, E-08003 Barcelona, Spain, ^b^Pharmaceutical Department, University of Parma Viale G. Usberti 27/A, 43100, Parma, Italy, ^c^Computational Biochemistry and Biophysics Laboratory, Research Unit on Biomedical Informatics (GRIB), Universitat Pompeu Fabra, Dr Aiguader 88, E-08003 Barcelona.*

**Content**

1. **Supplemental Experimental Procedures**
   1. Homology Modeling
   2. Validation of the Homology Modeling by Docking Studies
   3. Construction of the Receptor-Membrane Systems
   4. Molecular Dynamic Protocol
   5. Kernel Density Functions and Cluster Analysis
   6. Electron Density Profile Scheme
   7. Structural Prediction Protocol
2. **Supplemental References**

**1. Supplemental Experimental Procedures**

**1.1 Homology Modeling**

The 3D structure of the mGluR2 receptor was built by using the structure of the bovine rodhopsin (1GZM) as template and the sequence of the human mGluR2 receptor (**Q14416). With respect to our previous model of the mGluR2 receptor,** we used a new alignment (Figure S9). Comparing the new alignment with the old one, the main change refers to the TM5, where we shifted the alignment of four residues. In Figure S10, we report the comparison of the old and the new alignments for the TM5 domain. Finally, the new alignment was compared with the information available in literature [1,2].

sp|P02699|OPSD_BOVIN MNGTEGPNFYVPFSNKTGVVRSPFEAPQYYLAEPWQFSMLAAYMFLLIMLGFPINFLTLY

sp|Q14416|GRM2_HUMAN ---------------------------------IRWGDAWAVGPVTIACLGALATLFVLG

sp|P02699|OPSD_BOVIN VTVQHKKLRT--PLNYILLNLAVADLFMVFGGFTTTLYTSLHGYFVFGPTGCNLEGFFAT

sp|Q14416|GRM2_HUMAN VFVRHNATPVVKASGRELCYILLGGVFLCYCMTFIFIA----KPST---AVCTLRRLGLG

sp|P02699|OPSD_BOVIN LGGEIALWSLVVLAIERYVVVCKPMSNFRFG----------ENHAIMGVAFTWVMALACA

sp|Q14416|GRM2_HUMAN TAFSVCYSALLTKTNRIARIFGGAREGAQRPRFISPASQVAICLALISGQLLIVVAWLVV

sp|P02699|OPSD_BOVIN APPLVGWSRYIPEGMQ------CSCGIDYYTPHEETNNESFVIYMFVVHFIIPLIVIFFC

sp|Q14416|GRM2_HUMAN EAPGTGKE----TAPERREVVTLRCNHR----------DASMLGSLAYNVLLIALCTLYA

sp|P02699|OPSD_BOVIN YGQLVFTV-KEAAAQQQEATTQKAEKEVTRMVIIMVIAFLICWLPYAGVAFYIFTHQGSD

sp|Q14416|GRM2_HUMAN FKTRK----------CPENF------NEAKFIGFTMYTTCIIWLAFLPIFYVTSSDYRVQ

sp|P02699|OPSD_BOVIN FGPIF-MTIPAFFAKTSAVYNPVIYIMMNKQFRNCMVTTLCCGKNPLGDDEASTTVSKTE

sp|Q14416|GRM2_HUMAN TTTMCVSVSLSGSVVLGCLFAPKLHIILFQPQKNVVSHRAPT--SRFGSAAARASSSLGQ

sp|P02699|OPSD_BOVIN TSQVAPA

sp|Q14416|GRM2_HUMAN GSG----

**Figure S9. Human mGluR2-bovine rhodopsin alignment.** Highlighted in red the TM regions, in green the super-conserved residues for class A GPCRs on each TM, in yellow the residues forming the allosteric binding pocket for the RO4988546 and RO5488608 compounds.


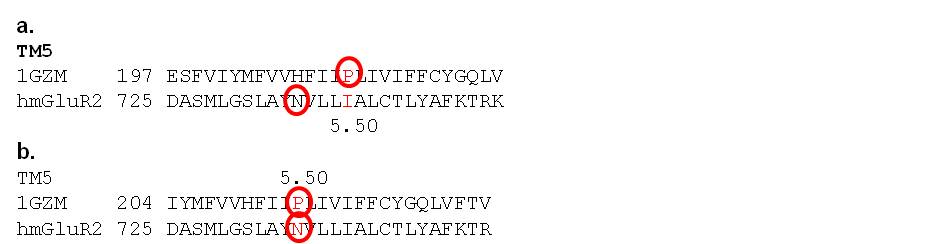


**Figure S10. Alignment comparison.** Comparison of the new **(A)** and the old **(B)** alignment for the TM5.

The 3D structure of the mGluR2 was generated using the MODELLER software (salilab.org/modeller) which carried out 10 models. The best model was selected among these 10 structures according to the MODELLER objective function and visual inspection. We evaluated the quality of the model on the basis of structural properties such as the presence of the conserved ionic lock and the presence of the disulphide bond between C^3.25^ and C^EL2.50^. In Figure S10A and S10B, we report the selected model of the mGluR2 receptor. It can be appreciated the presence of the disulphide bond (Figure S11A) and a tight network of interaction, in the bottom part of the receptor, involving the E^2.42^; K^3.46^; R^3.49^ and E^6.33^ (Figure S11B).


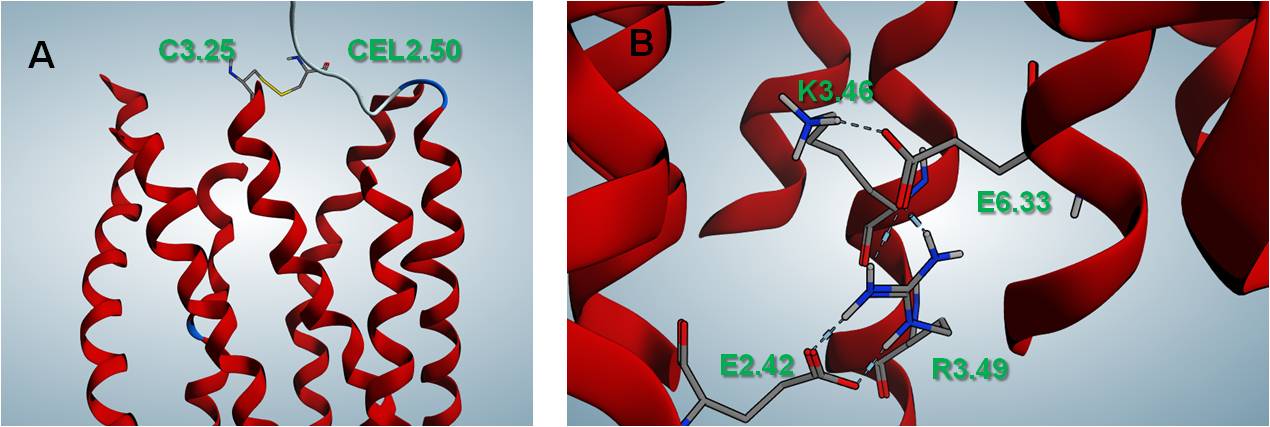


**Figure S11. Analysis of the structural properties of the generated mGluR2 model. (A)** Disulphide bond between the residues of C^3.25^ and C^EL2.50^; **(B)** ionic interactions at the bottom part (intracellular end of the TM domains) of the mGluR2 receptor.

**1.2 Validation of the Homology Model by Docking Studies**

To evaluate the structural architecture of the obtained mGluR2 model in more detail, two recently reported mGluR2 negative allosteric modulators, RO4988546 and RO5488608 (Chart 1), were docked into the model of TM-mGluR2 (Figure S12A-D). Importantly, the ligand-receptor interactions are in good agreement with mutagenesis data reported by [2] (Figure S12C,D) stressing the biological relevance of mGluR2 model. Recently, [2] identified by site-directed mutagenesis eight residues involved in the binding of two negative allosteric modulators for the mGluR2 receptor. According to these data, compounds RO4988546 and RO5488608 (Chart 1) share a common binding pocket defined by the R^3.28^, R^3.29^, F^3.36^, H^EL2.52^, L^5.43^, W^6.48^, F^6.55^, V^7.43^ residues. In order to validate our new model based on the rhodopsin template we performed a preliminary docking study by using the same compounds (RO4988546 and RO5488608) and the information available in the work of [2]. Differently to the work of [2] where compounds were manually docked, we performed docking studies by using the Glide induced fit docking (IFD) tool available in Maestro9.0 (Schrödinger, Mestro version 9.0 Schrödinger, LLC, New York 2009). The grid was centered on the residues shaping the putative allosteric binding pocket of the mGluR2 receptor (R^3.28^, R^3.29^, F^3.36^, H^EL2.52^, L^5.43^, W^6.48^, F^6.55^, V^7.43^), the flexible region was fixed until 5 Å around the center of the grid. Each docking run was carried out with the standard precision (SP) method, and the van deer Waals scaling factor of non polar atoms was set to 0.8. For each compound we used the same IFD protocol that provided us with 15 docking poses per compound. Among these poses we selected the best pose in accordance with the mutagenesis data. After that each complex ligand-receptor was subjected to minimization by using the MMFF94x available in MOE (http://www.chemcomp.com/), until a gradient of 0.005 Kcal/mol*Å^2^.


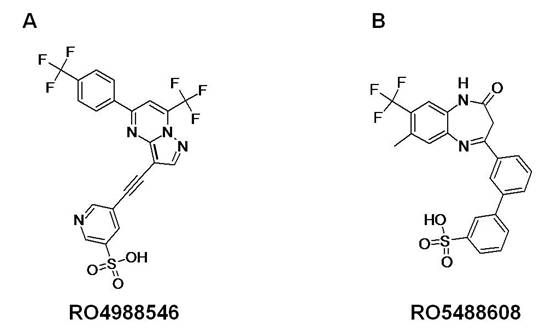


**Chart S1. Representation of the 2D structures of the compounds used for the docking studies.** Compounds used for the docking studies on the mGluR2 receptor. RO4988546 (**A**) and RO5488608 (**B**).

**
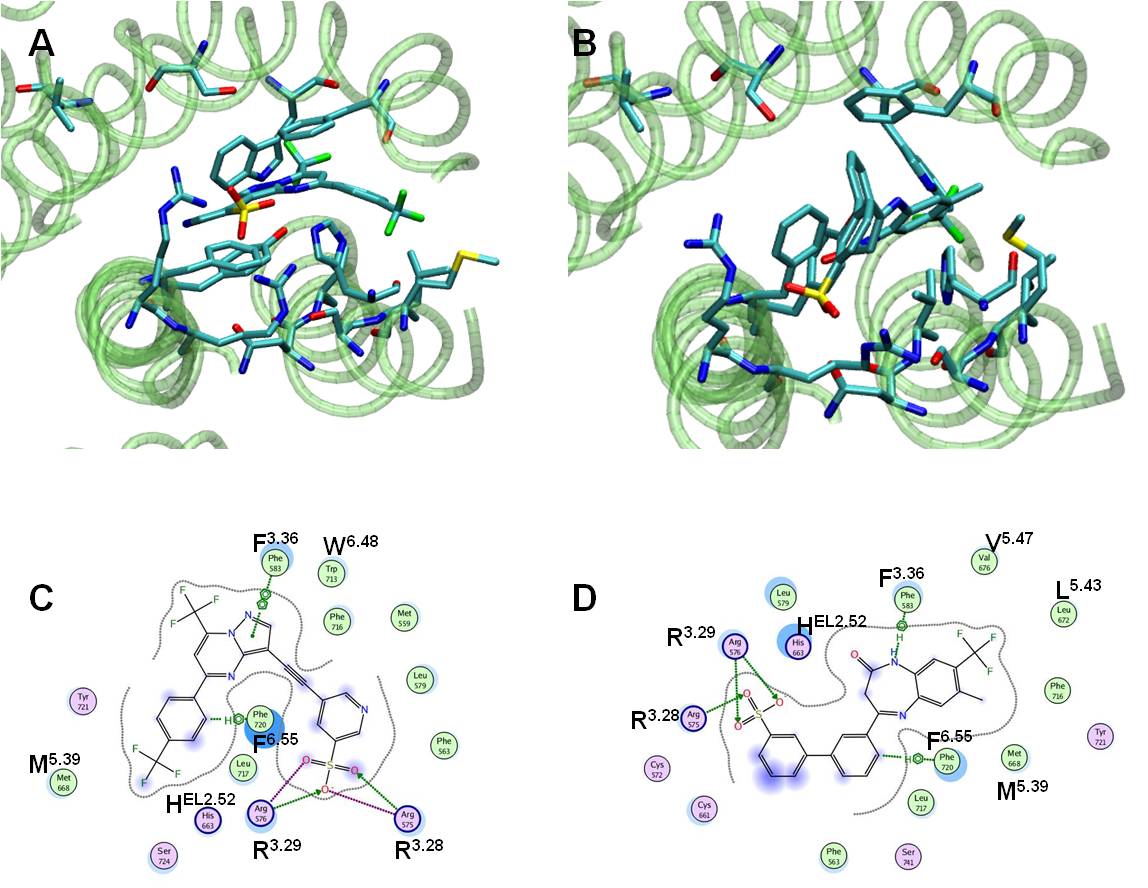
**

**Figure S12. Analysis of the docking studies.** Representation of the docking pose for the RO4988546 (**A**) and RO5488608 (**B**) compounds and the ligand-receptor interaction C and D respectively.

**1.3 Construction of the Receptor-Membrane-Cholesterol System.**

Two different pre-equilibrated SDPC phospholipids bilayers were generated using the membrane-builder tool of charm-gui.org (http://www.charmm-gui.org) (*i)* 94x94 Å (xy) 0% of cholesterol; (*ii*) 94x94 Å (xy) with a ratio SDPC:cholesterol equal to 1:3. In order to place the receptor into the bilayer a hole was generated, and lipids in close contact (<1 Å distance from any protein atoms) were deleted. For the membrane with cholesterol some SDPC or cholesterols molecule were manually deleted in each layer in order to retain the same cholesterol concentration (25%). The membrane-receptor complexes thus obtained were solvated and neutralized using the solvation and autoionize modules of VMD1.8.7 [3]. The ionic strength was kept at 0.15 M by NaCl and we used TIP3 water model. The all-atom models of each system were generated by using the CHARMM force-field parameters (Table S2) (http://mackerell.umaryland.edu/CHARMM_ff_params.html).

**Table S2. Structural details of the generated receptor-membrane complexes.** Final Structural Properties of the Membrane-Receptor Complex.

| **Membrane-Receptor Complex** | | | | | | | |
| --- | --- | --- | --- | --- | --- | --- | --- |
| **mGluR2** | | | | | | | |
| **0%**  **Chol** | | | | **25%**  **Chol** | | | |
| **System Size**  **(Å)** | | | | **System Size**  **(Å)** | | | |
| ***X*** | ***Y*** | | ***z*** | ***x*** | ***y*** | | ***z*** |
| 94 | 94 | | 110 | 94 | 94 | | 110 |
| **Number of Lipids** | | | | **Number of Lipids** | | | |
| 200 SDPC | | | | 60 Chol  /  180 SDPC | | | |
| **Ions** | | | | **Ions** | | | |
| **SOD** | | **CLA** | | **SOD** | | **CLA** | |
| 44 | | 60 | | 44 | | 60 | |
| **Waters** | | | | **Waters** | | | |
| 18231 | | | | 18282 | | | |
| **Box Type** | | | | **Box Type** | | | |
| Rectangle | | | | Rectangle | | | |

**1.4 Molecular Dynamics Protocol**

Before the relaxation step each system was submitted to a minimization procedure for 1000 steps. During the relaxation phase the system were equilibrated using the NPT ensemble with a target pressure equal to 1.01325 bar, a time-step of 2 fs and using the RATTLE algorithm for the hydrogen atoms. In this stage, the harmonic constraints were progressively reduced until an elastic constant force equal to 0 kcal/mol (step2 relaxation phase Table S3) and the temperature was increased to 300K. All the simulations were conducted using the same non-bonded interaction parameters, with a cutoff of 9 Å, a smooth switching function of 7.5 Å and the non-bonded pair list set to 9.5 Å. The periodic boundary conditions were set by using the system size shown in Table S2, and for the long range electrostatics we used the PME methodology with a grid spacing of 1 Å [4]. Each production phase was performed using the same parameters, with a time-step of 4 fs, and a hydrogen scaling factor of 4.

**Table S3. MD parameter settings.** Parameters used for the MD equilibration and production phases.

| **Relaxation Phase** | | | | | | | | | |
| --- | --- | --- | --- | --- | --- | --- | --- | --- | --- |
|  | **Timestep** | **Numstep** | **Temperature** | **Harmonic Constraints**  **(kcal/(mol*Å^2^))** | | | | | **Constant Temperature control** |
|  | | | | ***Backbone*** | ***Sidechains*** | ***Lipids*** | ***Waters*** | ***Ions*** |  |
| ***Step1*** | 2.0 fs | 10 ns | 200 K | 10 | 10 | - | - | - | NPT |
| ***Step2*** | 2.0 fs | 10 ns | 300 K | - | - | - | - | - | NPT |
| **Production Phase** | | | | | | | | | |
| ***Step1*** | 4.0 fs | 160 ns | 300 K | **-** | **-** | **-** | **-** | **-** | NPT |

**1.5 Kernel Density Function and Cluster Analysis**

To support the reliability of the results about the H8 stability obtained in a 160 ns MD run for the mGluR2 receptor with and without cholesterol, we performed a statistical analysis based on 10 MD runs for both simulations. Each MD runs was performed using the same experimental condition of the production phase (see Table S3). After that for each system with and without cholesterol, the 10 MD runs were concatenated in two whole trajectories, with and without cholesterol, respectively. A probability density function (PDF) was estimated using a two dimensional space defined by the values of the radius of gyration (G_(r)_) and the RMSD of the H8 from the starting structure. To build the representation spaces we concatenated the trajectories in two different ways, sampling the frames at intervals of 1 and 0.5 ns, for both system with and without cholesterol (Figure S4A-C and Figure S5A-D). The PDF was estimated building a Bivariate Kernel Density Function [5,6]. Kernel Density Estimation (KDE) is a non-parametric way of estimating the PDF of random variable. KDE is a fundamental data smoothing problem where inferences about the population are made, based on a finite data sample. To compute the PDF we used the Free Statics and Forecasting Software Server, based on R language (http://www.wessa.net/rwasp_bidensity.wasp). PDF is strongly affected by the bandwidth selection, and the use of Free Statics and Forecasting Software Server allows us to appropriately choose the bandwidth matrix H, in this case to estimate H the simple covariance matrix was used. In Figure 1 and 2 (see main text) we plotted each frame on the base of the PDF computed using the KDE on a bidimensional space defined by the G_(r)_and the RMSD of the H8 from the starting structure. The contour plot represents the standard density estimate and its bandwidth. The numbers beside the counter plot described the density estimation for that state. Higher is the number, greater is the probability to find that state. This analysis provides a relative estimation of the PDF to find some states in our simulation, because of it is based on the finite data sample described by our MD runs. Finally, to define the representative structures of each probable state found with the analysis of the PDF we clusterized the concatenated trajectories according to the RMSD and G_(r)_of the backbone atoms of the residues forming the putative H8. The cut off values used to clusterize the MD runs are reported in Table S4A and B. For each cluster a representative structure of the cluster was obtained using the *ptraj tool* of Amber 11 and the average linkage algorithm (http://ambermd.org/).

**Table S4. Cut-off used for the cluster analysis.**

**A.**

| **Cluster** | **RMSD**  **cut off** | **G_(r)_**  **cut off** |
| --- | --- | --- |
| **1** | <1 | > 6.5 |
| **2** | 1.2-2.2 | < 6.8 |
| **3** | 2.7-3.6 | > 6.8 |
| **4** | 3-3.5 | < 6.4 |
| **5** | > 3.5 | < 6.1 |

**B.**

| **Cluster** | **RMSD**  **cut off** | **G_(r)_**  **cut off** |
| --- | --- | --- |
| **1** | 0.5-1.5 | 6.5-7.4 |
| **2** | 1.5-2.5 | 6.3-7.0 |

**1.6 Electron Density Profile Scheme**

It has been reported that the presence of cholesterol can modify the thickness of a lipid bilayer [7,8]. To quantify the membrane thickness, we computed the local 1-D electron density profile of different species and functional groups (such as the entire SDPC and cholesterol molecules or the PO_4_ groups of the SDPC molecules) projected along the bilayer normal. The density profile computations were performed with the VMD plugin *Density Profile Tool* (http://multiscalelab.org/utilities/DensityProfileTool) as follows: first, the simulation box is partitioned into several parallel slabs, measuring Δz = 1Å along the z axis; the slabs extend up to the boundaries of the simulation box along x and y (Figure S13). The approximate number of electrons within each slab is then computed, for each simulation frame, summing the atomic number, Z, and the partial charge, δ, of the atoms whose centers are located inside each slab. The total is finally normalized by the slab volume, so that the local electron density at *z,* expressed in units of electrons/Å^3^ , is as follows:


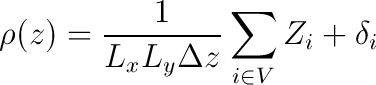


where Δz is the slab thickness (profile resolution), Lx and Ly are the simulation box dimensions orthogonal to the z axis, V_z_ is the set of atoms whose centers lie inside the slab centered at z and Δz thick. We assumed the membrane thickness to be the peak-to-peak distance (in Å) in the density profile plot.


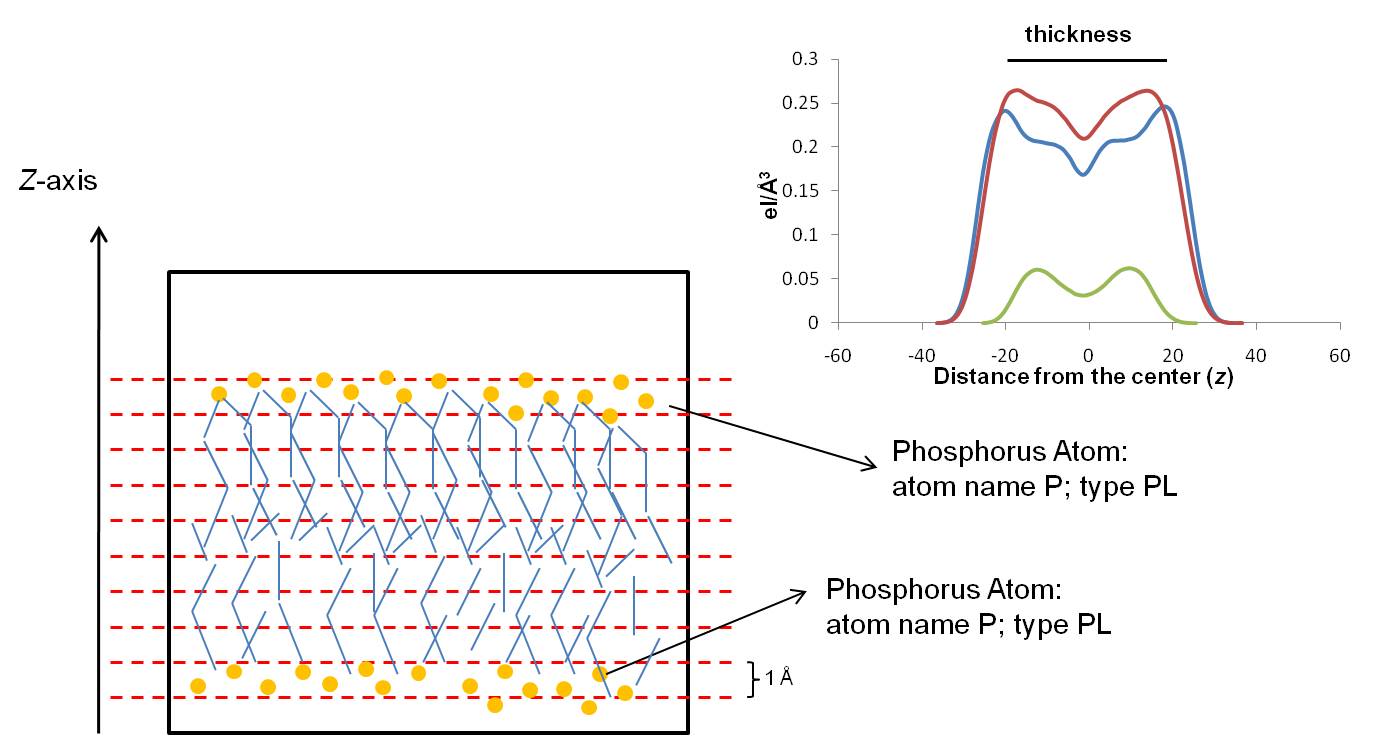


**Figure S13. EDP scheme.** At each simulation frame**,** the EDP is computed by summing the atomic number (Z) and the partial charges of the atoms falling into 1 A-thick slabs parallel to the z axis. The sum, normalized by volume, provides the local 1-D density value around z.

The system with cholesterol, shows a peak-to-peak distance (equal to bilayer thickness) of 36 Å (Figure S6A, blue line) in which each peak refers to the EDP of carbonyl group of the lipid tails with an experimental value of z= 18.4 Å [9]. The system without cholesterol adopts an average peak-to-peak distance of 30 ± 1.15 Å (Figure S6A, red line). These data are in good agreement with experimental data [9–13].

Interestingly, the EDP membrane plot (Figure S6B) shows also a slight asymmetric behavior. This is most likely due to (*i*) a different number of SDPC and cholesterol molecules for each layer (extracellular layer: 87 SDPC and 29 cholesterol molecules; intracellular layer 93 SDPC and 31 cholesterol molecules) [14–17]; (*ii*) an asymmetric shape of the mGluR2.

**Average values:** so as to compute the average values of the bilayer thickness and the location of the hydrophobic face of the H8, for each MD run we computed its own PO_4_ and H8 (only the Cα of the residue lining up the hydrophobic face) EDP profiles. The average values were computed as follows:

1. from each MD run we extrapolated the EDP values of each slab
2. The EDP values at the corresponding slab (distance from the center along the z axes) were averaged along the ten MD runs. (For each slab we have ten values)
3. For each average value at that slab we computed the standard deviation parameters
4. Finally, in Figure 2A and D (see main text) we plotted the average EDP profile for the membrane and the H8, with its cognate standard deviations.

The average thickness values were computed as follows:

1. For each MD run we computed the thickness
2. The average thickness and its cognate standard deviations values were computed using the ten MD runs for each system, with and without cholesterol.

The H8 Hydrophobic phase was computed using as reference the Cα atoms of the H8 facing the membrane bilayer, the average plot reported in Figure 2B and 2E (see main text) was computed as described above.

Since the distance between the peak of the PO_4_ groups and the hydrophobic face of the H8 represents the most intriguing results of our study, we need to establish the exact amount of this value with particular precision. Thus, the distance between the peak of the PO_4_ groups and the hydrophobic face of the H8 was computed as follows:

1. In each MD run the peak values showed certain degree of uncertainty, therefore in each MD run we identified the exact location (the exact slab where the peak is located) of the peak for both PO_4_ and hydrophobic face of the H8
2. The average locations were computed using the exact slab values extrapolated from the analysis aforementioned, we also computed the standard deviation of the average location (Figure 2A and 2D, main text).

The distance of the PO_4_ and hydrophobic face of the H8 peaks was computed using these values (Figure 2A and 2D, main text).

**1.7 Structural Prediction Protocol**

The amphipathic character of the putative mGluR2 H8 was assessed by AmphipaSeek, along with an evaluation of the potential presence of in-plane membrane (IPM) anchor points, an structural motif of amphipathic helices (Figure S7 and S8). However, AmphipaSeek did not identify any IPMs in mGluR2, human CXCR, as well as bovine rhodopsin although it is known that bovine rhodopsin has an amphipathic in-plane α-helix, with two palmitylation sites [18,19]. Moreover, applying AmphipaSeek for computing the amphipathicity and the hydrophobic moment yielded only inconsistent patterns when comparing GPCRs with/without conserved H8 (Figure S8). Hence, AmphipaSeek is not a suitable tool for detecting amphipathic H8 domains in GPCRs.

To support the genuine occurrence of an α-helical H8, we screened the Protein Data Bank using BLAST (http://blast.ncbi.nlm.nih.gov/Blast.cgi) for sequences homolog to the mGluR2-H8 sequence (ILFQPQKNV) (Table 1 main text). In a first step, we validated our search protocol by using the corresponding H8 sequences of the bovine rhodopsin and CXCR4 receptors (Table 1 main text) taking into account only the first three proteins that were retrieved by our search protocol and that were not X-ray crystal structures of the same receptor (Table 1 main text). Applying this protocol, we were able to predict an α-helical structure for the H8 of the bovine rhodopsin receptor which is in accordance with the X-ray structure. More intriguing, we were able to identify successfully the non α-helix region of the CXCR4 (Table 1 main text, 2JRC.pdb) as well as the short region with an α-helical structure AQHAL (Table 1 main text, 1SJ8.pdb and 2BEK.pdb). In a second step, the same search protocol was applied to the corresponding H8 sequence for the mGluR2 receptor (Table 1 main text). Thereby, the search retrieved a X-ray template for the sequence fragment ILFQ adopting an α-helical structure in 3EFO.pdb and 1IC8.pdb. In addition, for the sequence fragment QKNVVSHR, we came across a highly homolog template (EKNAVSHR) in the 215D.pdb X-ray structure forming a α-helix.

Taking the experimental and computational results together, we propose that the mGluR2 possesses an amphipathic H8 which can adopt an α-helical conformation depending on the membrane environment which is an issue of high relevance for G protein binding and receptor functionality.

**2. Supplemental References**

1. Binet V, Duthey B, Lecaillon J, Vol C, Quoyer J, et al. (2007) Common Structural Requirements for Heptahelical Domain Function in Class A and Class C G Protein-coupled Receptors. J. Biol. Chem. 282: 12154 –12163. doi:10.1074/jbc.M611071200.

2. Lundström L, Bissantz C, Beck J, Wettstein JG, Woltering TJ, et al. (2011) Structural determinants of allosteric antagonism at metabotropic glutamate receptor 2: mechanistic studies with new potent negative allosteric modulators. Br. J. Pharmacol. 164: 521-537. doi: 10.1111/j.1476-5381.2011.01409.x.

3. Humphrey W, Dalke A, Schulten K (1996) VMD: visual molecular dynamics. J. Mol. Graph. 14: 33–38, 27–28.

4. Darden T, York D, Pedersen L (1993) Particle mesh Ewald: An N⋅log(N) method for Ewald sums in large systems. J. Chem. Phys. 98: 10089. doi:10.1063/1.464397.

5. Sheather SJ, Jones MC (1991) A Reliable Data-Based Bandwidth Selection Method for Kernel Density Estimation. Journal of the Royal Statistical Society Series B (Methodological) 53: 683–690.

6. Yeung KY, Fraley C, Murua A, Raftery AE, Ruzzo WL (2001) Model-based clustering and data transformations for gene expression data. Bioinformatics 17: 977–987.

7. Olsen BN, Schlesinger PH, Baker NA (2009) Perturbations of Membrane Structure by Cholesterol and Cholesterol Derivatives Are Determined by Sterol Orientation. J. Am. Chem. Soc. 131: 4854–4865. doi:10.1021/ja8095224.

8. Eriksson ESE, Eriksson LA (2011) The Influence of Cholesterol on the Properties and Permeability of Hypericin Derivatives in Lipid Membranes. J. Chem. Theory Comput. 7: 560–574. doi:10.1021/ct100528u.

9. Mihailescu M, Soubias O, Worcester D, White SH, Gawrisch K (2010) Structure and Dynamics of Cholesterol-Containing Polyunsaturated Lipid Membranes Studied by Neutron Diffraction and NMR. J. Membr. Biol. 239: 63–71. doi:10.1007/s00232-010-9326-6.

10. Binder H, Gawrisch K (2001) Effect of Unsaturated Lipid Chains on Dimensions, Molecular Order and Hydration of Membranes. J. Phys. Chem. B 105: 12378–12390. doi:10.1021/jp010118h.

11. Gamba Z (2008) Effective potentials and electrostatic interactions in self-assembled molecular bilayers II: The case of biological membranes. J. Chem. Phys. 129: 215104. doi:10.1063/1.3026662.

12. Koenig BW, Strey HH, Gawrisch K (1997) Membrane lateral compressibility determined by NMR and x-ray diffraction: effect of acyl chain polyunsaturation. Biophys. J. 73: 1954–1966. doi:10.1016/S0006-3495(97)78226-2.

13. Saiz L, Klein ML (2001) Structural Properties of a Highly Polyunsaturated Lipid Bilayer from Molecular Dynamics Simulations. Biophysi. J. 81: 204–216. doi:10.1016/S0006-3495(01)75692-5.

14. Kučerka N, Nieh M-P, Katsaras J (2009) Asymmetric Distribution of Cholesterol in Unilamellar Vesicles of Monounsaturated Phospholipids. Langmuir 25: 13522–13527. doi:10.1021/la9020299.

15. Rukmini R, Rawat SS, Biswas SC, Chattopadhyay A (2001) Cholesterol organization in membranes at low concentrations: effects of curvature stress and membrane thickness. Biophys. J. 81: 2122–2134. doi:10.1016/S0006-3495(01)75860-2.

16. Mannock DA, Lewis RNAH, McMullen TPW, McElhaney RN (2010) The effect of variations in phospholipid and sterol structure on the nature of lipid-sterol interactions in lipid bilayer model membranes. Chem. Phys. Lipids 163: 403–448. doi:10.1016/j.chemphyslip.2010.03.011.

17. Patel RY, Balaji PV (2008) Characterization of Symmetric and Asymmetric Lipid Bilayers Composed of Varying Concentrations of Ganglioside GM1 and DPPC. J. Phys. Chem. B 112: 3346–3356. doi:10.1021/jp075975l.

18. Topiol S, Sabio M (2009) X-ray structure breakthroughs in the GPCR transmembrane region. Biochem. Pharmacol. 78: 11–20. doi:10.1016/j.bcp.2009.02.012.

19. Park PS-H, Sapra KT, Jastrzebska B, Maeda T, Maeda A, et al. (2009) Modulation of Molecular Interactions and Function by Rhodopsin Palmitylation. Biochemistry 48: 4294–4304. doi:10.1021/bi900417b.
